# Discovery of Pyrimidine- and Coumarin-Linked Hybrid Molecules as Inducers of JNK Phosphorylation through ROS Generation in Breast Cancer Cells

**DOI:** 10.3390/molecules28083450

**Published:** 2023-04-13

**Authors:** Na Young Kim, Divakar Vishwanath, Zhang Xi, Omantheswara Nagaraja, Ananda Swamynayaka, Keshav Kumar Harish, Shreeja Basappa, Mahendra Madegowda, Vijay Pandey, Gautam Sethi, Peter E. Lobie, Kwang Seok Ahn, Basappa Basappa

**Affiliations:** 1 Department of Science in Korean Medicine, Kyung Hee University, 24 Kyungheedae-ro, Dongdaemun-gu, Seoul 02447, Republic of Korea; nay0kim@naver.com; 2 Laboratory of Chemical Biology, Department of Studies in Organic Chemistry, University of Mysore, Manasagangotri, Mysore 570006, India; divakardivi166@gmail.com; 3 Shenzhen Bay Laboratory, Shenzhen 518055, China; zhangxi@szbl.ac.cn; 4 Department of Studies in Physics, University of Mysore, Manasagangotri, Mysore 570006, India; omantheswara@physics.uni-mysore.ac.in (O.N.); anandas@physics.uni-mysore.ac.in (A.S.); keshavkumar@physics.uni-mysore.ac.in (K.K.H.); mahendra@physics.uni-mysore.ac.in (M.M.); 5 Department of Chemistry, BITS-Pilani Hyderabad Campus, Jawahar Nagar, Medchal 500078, India; f20210833@hyderabad.bits-pilani.ac.in; 6 Tsinghua Berkeley Shenzhen Institute, Tsinghua Shenzhen International Graduate School, Tsinghua University, Shenzhen 518055, China; vijay.pandey@sz.tsinghua.edu.cn; 7 Institute of Biopharmaceutical and Health Engineering, Tsinghua Shenzhen International Graduate School, Tsinghua University, Shenzhen 518055, China; 8 Department of Pharmacology, Yong Loo Lin School of Medicine, National University of Singapore, Singapore 117600, Singapore; phcgs@nus.edu.sg

**Keywords:** JNK signaling, pyrimidine, coumarin, HER-2 positive breast cancer, apoptosis

## Abstract

Human epidermal growth factor receptor 2 (HER2)-positive breast cancer exhibits early relapses, poor prognoses, and high recurrence rates. Herein, a JNK-targeting compound has been developed that may be of utility in HER2-positive mammary carcinoma. The design of a pyrimidine-and coumarin-linked structure targeting JNK was explored and the lead structure PC-12 [4-(3-((2-((4-chlorobenzyl)thio) pyrimidin-4-yl)oxy)propoxy)-6-fluoro-2*H*-chromen-2-one (**5d**)] was observed to selectively inhibit the proliferation of HER2-positive BC cells. The compound PC-12 exerted DNA damage and induced apoptosis in HER-2 positive BC cells more significantly compared to HER-2 negative BC cells. PC-12 induced PARP cleavage and down-regulated the expression of IAP-1, BCL-2, SURVIVIN, and CYCLIN D1 in BC cells. In silico and theoretical calculations showed that PC-12 could interact with JNK, and in vitro studies demonstrated that it enhanced JNK phosphorylation through ROS generation. Overall, these findings will assist the discovery of new compounds targeting JNK for use in HER2-positive BC cells.

## 1. Introduction

A hierarchical clustering analysis has classified breast cancer into distinct molecular subtypes: luminal A/B, basal, normal, and HER2-positive. Anti-HER2 monoclonal antibodies such as trastuzumab and targeted small molecules may be used to treat HER-2 positive breast cancer, however many challenges remain to achieve optimal clinical outcomes for the HER2 subtype [1,2,3]. These include HER-2 therapy resistance and relapse, with high recurrence rates and poor prognoses [4]. With understanding of the downstream signaling of HER2, more effective therapies can be designed to potentially overcome resistance. JNK signaling is one such potential target and SP600125, a pan-JNK inhibitor, effectively inhibited HER2-positive and resistant mammary carcinoma [5].

**Figure 1:**
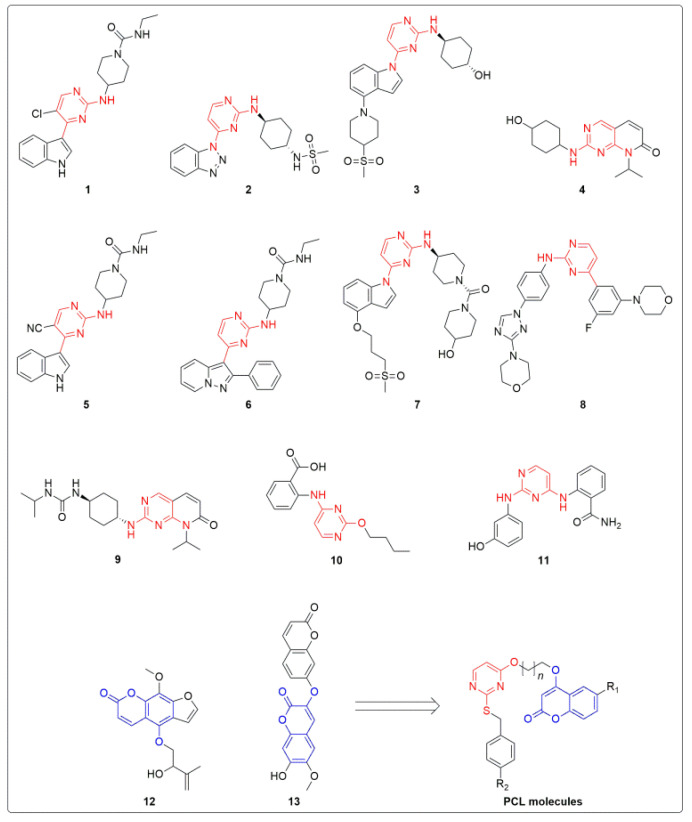
Evolution of newer pyrimidine-linked coumarins (PCLs) as novel chemical entities that target JNK in BC cells.

Based on the availability of inhibitors that target c-Jun N-terminal kinase (JNK), the compounds may be classed into various groups, such as open and closed conformation binders, gatekeeper residue binders, peptide-based inhibitors, covalent inhibitors, and inhibitors of type II kinases [6]. Structure-based analysis herein revealed that most of the co-crystal structures of ligands and JNK includes aminopyrimidines as a central scaffold, where most aminopyrimidines bind to the ATP pocket of JNK containing the closed conformation of Met146, which is responsible for the interaction of all JNK isoforms [7]. According to X-ray crystallography, the indazole–aminopyrimidine compound binds to JNK1b (PDB: 4HYU) and interacted via two hydrogen bonds with the amino acid backbones of the hinge region [8]. A co-crystal structure of JNK3 (PDB: 3KVX) and the aminopyridine ligand revealed that it was positioned within the adenosine-binding region, and the morpholino substituent was positioned within the hydrophilic sugar pocket [9]. It was also observed in a co-crystal structure of JNK3 (PDB: 4Y5H) that two hydrogen bonds with Met149 were formed at the adenine-binding site [10]. Furthermore, pyrimidine-based ligands and the crystal structure of JNK1 (PDB: 2NO3) revealed that two hydrogen bonds formed with the hinge region Met111 [11]. In the search for a novel JNK inhibitor, chemical structures 1–11 were potent, selective, and orally active inhibitors. In addition to the aminopyridine scaffold, coumarin-based heterocycles, such as compounds 12 (neobyakangelicol) and 13 (daphnoretin), were observed to be potent inhibitors of JNK (Figure 1) [12,13]. In this study, pyrimidine-linked coumarin with various substitutions was synthesized to develop a hybrid structure that could target JNK in BC cells.

## 2. Results and Discussions

### 2.1. Chemical Synthesis of PCLs (**5a–h**)

**Scheme 1:**
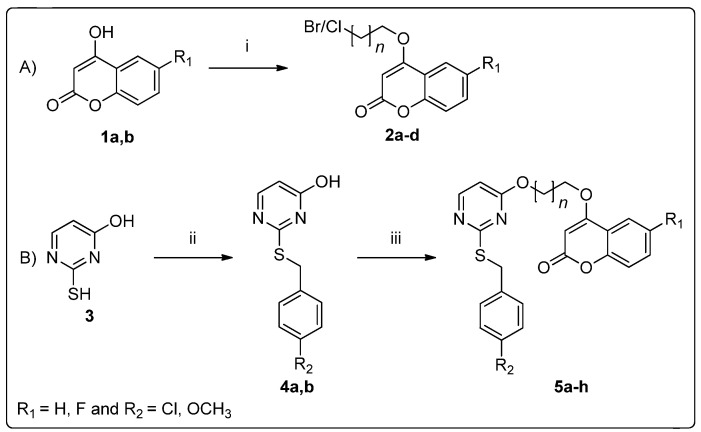
Schematic representation of the synthesis of title compounds (**5a**–**h**). A, synthesis of coumarin based alkyl halides (**2a**–**d**); B, reactivity representation of alkylated thio-uracil and (**2a**–**d**)**. *Reagents and conditions:*** (**A**)*,* (i) 1,2-dibromoethane/1-bromo-3-chloro propane, DMF, K_2_CO_3_, reflux; (**B)**, (ii) substituted benzyl bromides or benzyl chlorides, EtOH:H_2_O (1:1), KOH, 45 °C; (iii) (**2a**–**d**), acetone, K_2_CO_3_, reflux.

Recently, pyrimidine-based amides were developed as inhibitors of poly (ADP-ribose) polymerase, which may provide novel drug-seeds for the development of target-based drugs for BC [14]. Earlier, the synthesis of 4-fluoro-biphenyl linked 1,2,4-triazolo[1,5-*a*]pyrimidines via click chemistry was reported and it was observed that these small molecules targeted VEGFR1 in breast cancer cells [15]. Furthermore, coumarins were also synthesized, where the compound named CPP induced cytotoxicity in hepatocellular carcinoma (HCC) cells and suppressed the DNA binding ability of NF-κB [16]. In addition, it was observed that bis-coumarins bound to tumor necrosis factor-α and disrupted the native, trimeric structure of TNF, producing anti-inflammatory effects via the NF-κB-regulated pathway [17]. Herein, the target **PCLs** (**5a**–**h**) were synthesized via the reaction between substituted benzyl-2-thiouracils with alkylated coumarins in refluxing acetone under basic conditions (Scheme 1). TLC monitored the reaction completion, and the crude obtained was purified using column chromatography (hexane/ethyl acetate ratio of 70:30). The pure **PCLs** were characterized by NMR, mass spectrometry, and melting points. The structural and physical properties and IC_50_ of the **PCLs** are depicted in Table 1.

### 2.2. Efficacy of PCLs against a Variety of Breast Cancer Cells

**Figure 2:**
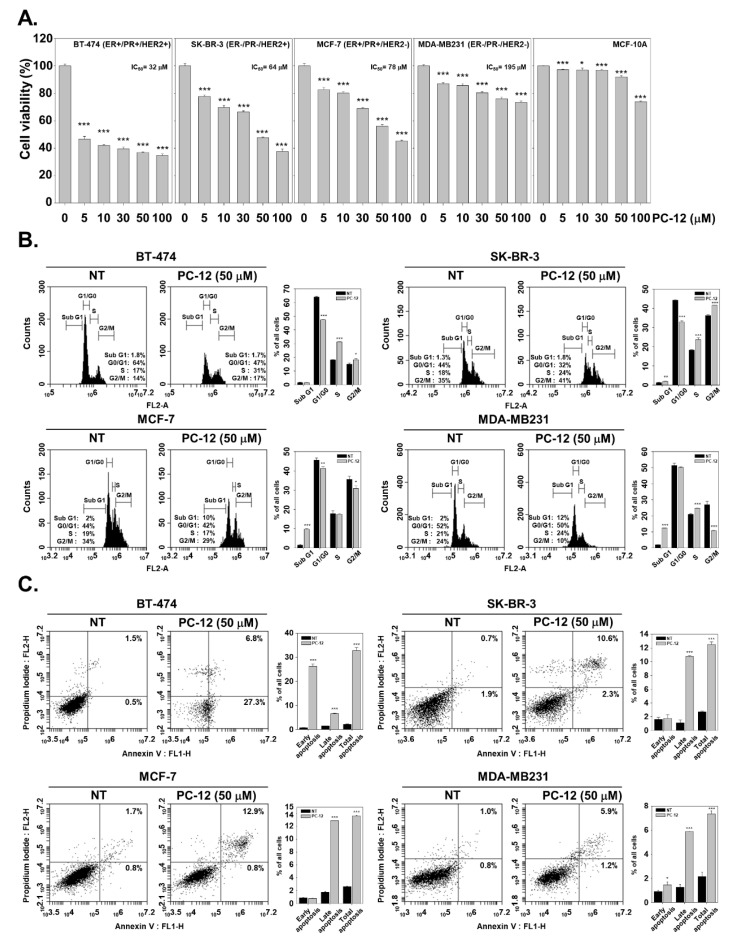
PC-12 produced loss of cell viability and induced cell cycle arrest. (**A**) MTT assay was performed for cell viability in PC-12-treated breast cell lines. BT-474, SK-BR-3, MCF-7, MDA-MB-231, and MCF-10A cells were treated with PC-12 (0–5–10–30–50–100 µM) for 24 h. *** *p* < 0.001 and * *p* < 0.05 vs. non-treated (NT) group. (**B**) Cells were exposed to PC-12 (0–50 µM) for 24 h, and cell cycle analysis was performed. *** *p* < 0.001, ** *p* < 0.01, and * *p* < 0.05 vs. non-treated (NT) group. (**C**) The four breast cancer cell lines were treated with 0–50 µM of PC-12 for 24 h, and cells were collected. The apoptotic cells were detected by flow cytometry using an Annexin/PI staining assay. *** *p* < 0.001 and * *p* < 0.05 vs. non-treated (NT) group.

The novel PCL compounds were initially subjected to cell viability studies against human breast cancer cells (MCF-7) using an MTT assay [18,19]. The positive controls, doxorubicin, and tamoxifen inhibited the proliferation of MCF-7 cells with IC_50_ values of 1.12 and 0.86 µM, respectively. The assay results show that compounds PC-09, PC-10, PC-11, PC-12, and PC-14 produced a loss of viability of MCF-7 cells with varying IC_50_ values of 31.29, 16.42, 47.22, 8.00, and 13.33 µM, respectively. The lead compound PC-12 was utilized for further characterization. Breast cancer cell lines of four different subtypes were used next. The loss of cell viability produced by PC-12 in triple-positive (ER+/PR+/HER2+; luminal A) BT-474 cells, HER2-positive SK-BR-3 cells (ER−/PR−/HER2+; HER2+), ER− and PR-positive MCF-7 cells (ER+/PR+/HER2−: Luminal A), triple-negative MDA-MB-231 cells (ER−/PR−/HER2−; representative of basal), and normal immortalized MCF-10A cells at different concentrations were confirmed by MTT assay (0, 5, 10, 30, 50, or 100 μM) for 24 h [20,21]. PC-12 exhibited the most potent loss of cell viability against BT-474 cells with an IC_50_ of 32 μM. The highest loss of cell viability was observed in SKBR-3 cells (Figure 2A). Also as shown in Figure 2A, triple-negative MDA-MB-231 cells exhibited minimal loss of cell viability in response to PC-12. In MCF-10A cells, PC-12 only produced a biologically significant loss of viability at 100 µM. Therefore, it was postulated that the compound PC-12 preferentially produced loss of cell viability in HER-2-positive BC cells.

### 2.3. PC-12 Induces Apoptosis in Human Breast Cancer Cells

Hybrid PCL molecules ave been reported as anticancer agents and found to induce G2/M arrest and apoptosis in MCF-7 cells [22]. Therefore, BC cell apoptosis produced by PC-12 was evaluated and which showed S-phase and G2/M-phase cell cycle arrest in BT-474 and SK-BR-3 cells, and an increased sub-G1 phase in MCF-7 and MDA-MB231 cells (Figure 2B). In addition, an Annexin/PI staining analysis for determination of apoptotic cells was performed. Total apoptosis in BT-474 cells increased from approximately 2% to 34%. In SK-BR-3 cells, late apoptotic cells were increased from 0.7% to 10.6% and in MCF-7 cells increased from 1.7% to 12.9%. In contrast, in MDA-MB-231 cells, total apoptotic cells increased from 1.0% to 5.9%, the lowest increase of the four cell lines examined (Figure 2C). These results confirm that PC-12 induced apoptosis in HER-2-positive BC cells preferentially compared to HER-2-negative BC cells.

### 2.4. PC-12 Promotes DNA Damage in Human Breast Cancer Cells

**Figure 3:**
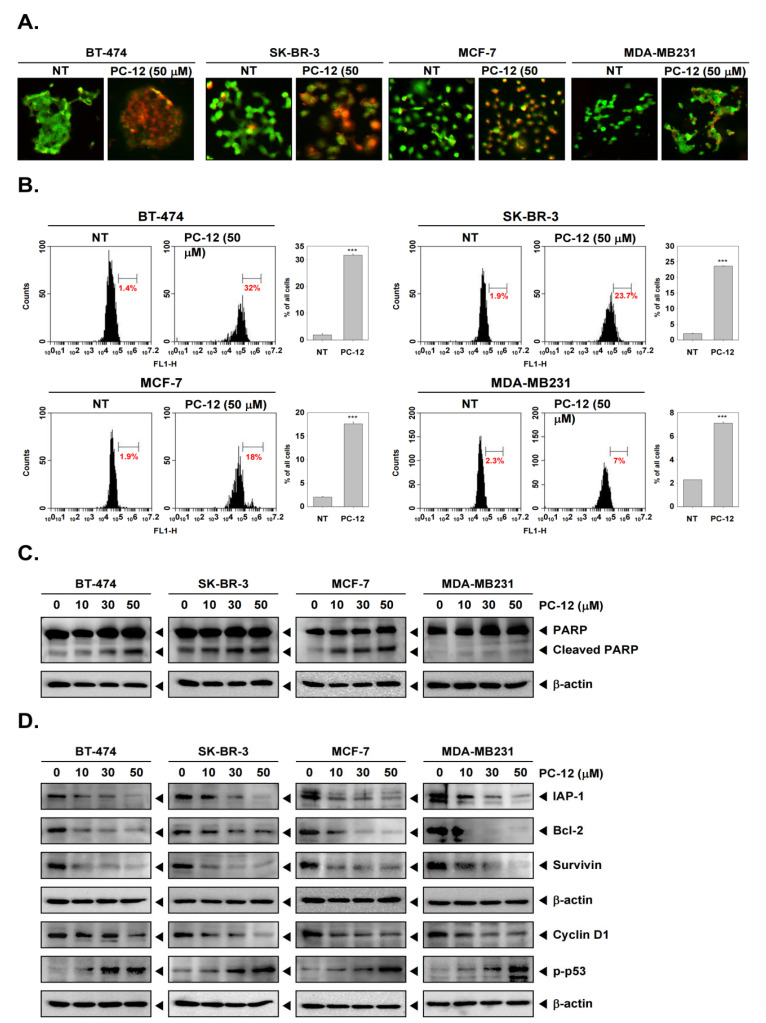
PC-12 produced DNA damage and modulated proteins related to apoptosis and cell survival. (**A**) The live and dead assay was performed using calcein AM and EthD-1. After 24 h treatment with PC-12 (0–50 µM), the cells were stained with Calcein AM and EthD-1 for 30 min at 37 °C, and then the live and dead assay results were determined by fluorescence microscopy. (**B**) The four cell lines were treated with the indicated concentrations of PC-12 for 24 h. TUNEL assay was performed to detect the induction of apoptosis. *** *p* < 0.001 vs. non-treated (NT) cells. (**C**,**D**) BT-474, SK-BR-3, MCF-7, and MDA-MB231 cells were incubated with 0, 10, 30 and 50 µM of PC-12 for 24 h. The cells were harvested and lysed, and then Western blot analysis was performed. β-ACTIN was used as input control.

A coumarin-based glycoside named esculin has been observed to inhibit oxidative DNA damage induced by 1,2-dimethylhydrazine in rat colon [23]. In general, increased nucleotide metabolism promoted the growth cancers. Drug seeds that inhibit nucleotide synthesis have also been used to reduce cancer growth, producing DNA damage and induced cell death. Therefore, it was examined whether PC-12 exerted a DNA-damaging effect on BC cells. Calcein AM and EthD-1 were used for the live and dead cell assay. Specifically, EthD-1 permeates damaged membranes and combines with nucleic acids in dead cells and emits red fluorescence [24]. As shown in Figure 3A,B, in response to PC-12, dead cells were the highest in BT-474 cells and the lowest in MDA-MB- 231 cells. Dead cells were also detected in SK-BR-3 and MCF-7 cells respectively. This data trend was consistent with the MTT assay and annexin/PI analysis. Hence, compound PC-12 produced DNA damage in BC cells.

### 2.5. PC-12 Induces Cleavage of PARP and Regulated the Expression of Apoptotic Proteins

A camphor-based pyrimidine has been reported to promote apoptosis associated with increased expression of pro-apoptotic proteins Bax, cytochrome C, and caspase-3 and decreased expression of anti-apoptosis protein Bcl-2 [25]. Herein, the effect of PC-12 on the expression of apoptotic genes and PARP cleavage (one of the hallmarks of apoptosis) was examined. PC-12 induced the cleavage of PARP in BT-474, SK-BR-3, and MCF-7 cells, but in MDA-MB-231 cells, PC-12 did not affect the levels of cleaved PARP (Figure 3C). In addition, PC-12 decreased the expression of IAP-1, BCL-2, SURVIVIN and CYCLIN D1 in these four cell lines; these proteins function in cell survival and proliferation [26]. Moreover, phosphorylation of p53 was enhanced with PC-12 treatment, which suggests that PC-12 inhibited cell survival and proliferation and induced apoptosis (Figure 3D).

### 2.6. PC-12 Induces Cell Death through ROS Production

**Figure 4:**
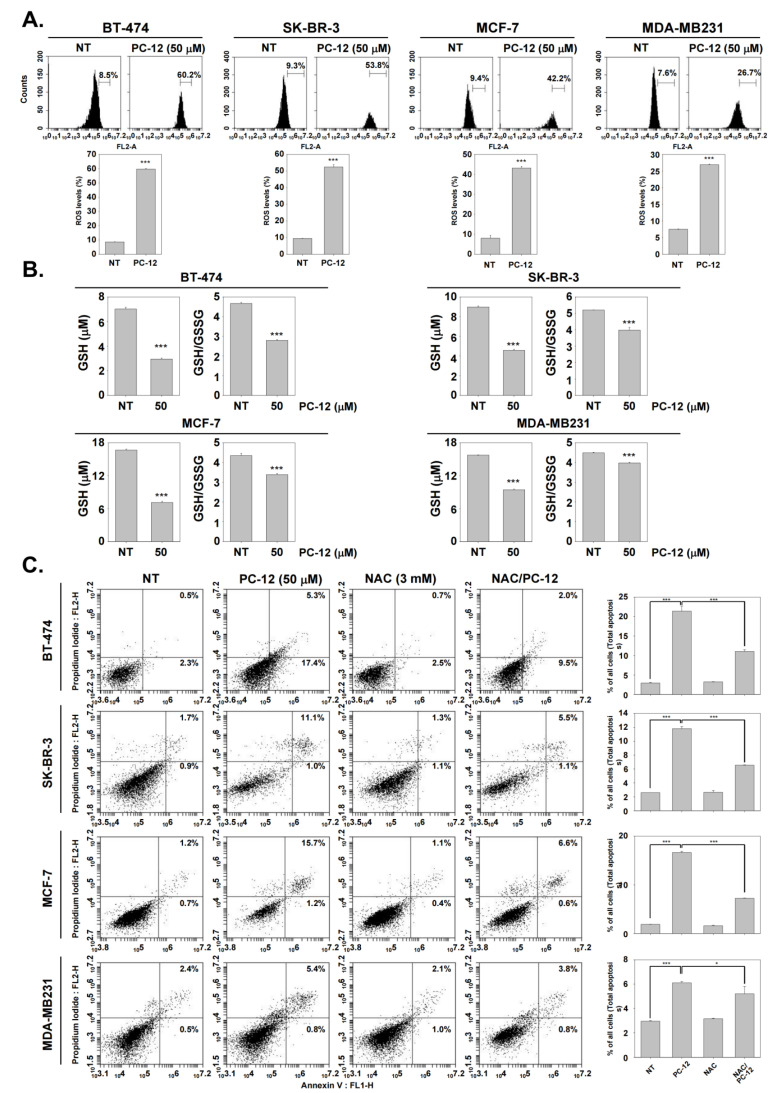
PC-12 induced apoptotic cells via ROS production. (**A**) PC-12 (0–50 µM) treatment in four cell lines for 12 h. The cells were collected, and ROS measurement was performed using H2DCFH-DA staining. *** *p* < 0.001 vs. non-treated (NT) cells. (**B**) PC-12 (0–50 µM) was incubated for 24 h with the four cell lines, and the GSH/GSSG assay was performed following the manufacturer’s protocol. *** *p* < 0.001 vs. non-treated (NT) cells. (**C**) BT-474, SK-BR-3, MCF-7, and MDA-MB231 cells were treated with 50 µM of PC-12 or 3 mM of NAC for 24 h, an Annexin/PI staining assay was performed to measure apoptotic cells. *** *p* < 0.001 and * *p* < 0.05 vs. non-treated (NT) group.

Many reports have stated that the reactive oxygen species (ROS) level is associated with apoptosis, and hence it was determined whether PC-12 increased ROS levels [27,28,29,30,31,32,33,34]. PC-12 increased ROS levels from 8.5% to 60.2% in BT-474 cells, 9.3% to 53.8% in SK-BR-3, 9.4% to 42.2% in MCF-7, and 7.6% to 26.7% in MDA-MB231 cells respectively (Figure 4A). Consistent with other assays above, PC-12 treatment induced ROS the most in BT-474 cells and the least in MDA-MB231 cells. In addition, GSH levels were decreased with PC-12 treatment in these four cells, and the GSH/GSSG ratio decreased (Figure 4B). Moreover, treatment with NAC, known as a ROS scavenger, reduced the number of apoptotic cells induced by PC-12. In BT-474 cells, the apoptotic cells accounted for approximately 22.7% with PC-12 treatment, but when combined with NAC total apoptotic cells accounted for only 11.5%. This trend was similar in other cells, which indicates that the compound **PC-12** induced cell death via ROS generation (Figure 4C).

### 2.7. Mechanism for ROS Generation by PC-12 via Frontier Molecular Orbital (FMO) Analysis

**Figure 5:**
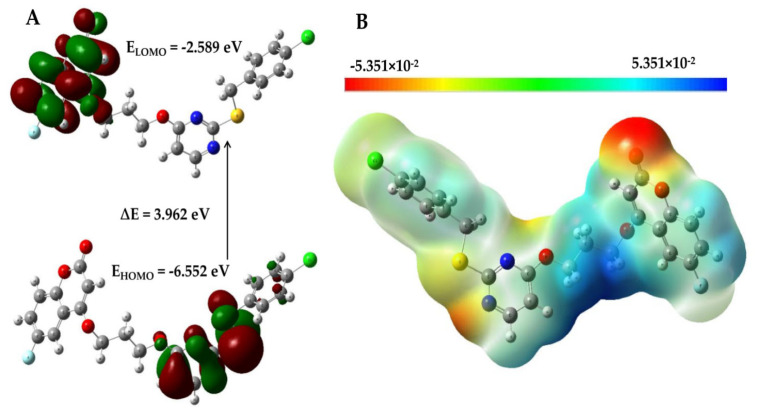
Computed molecular orbital (**A**) and electrostatic charge distribution (**B**) plots for PC-12. Colors on the surfaces of the atoms indicate different electrostatic potentials, with electrostatic potential value increasing in the following order: red < orange < yellow < green < blue.

By calculating the frontier molecular orbital energies of PC-12, the potential reaction pathways of ROS production could be measured. For this purpose, the B3LYP/6-311++G (d, p) level of theory was used to calculate the energy of the highest molecular orbital (HOMO), the lowest unoccupied molecular orbital (LUMO) energy, and the energy gap (∆E) estimated value in order to determine the redox potential of PC-12. The charge density distribution and energy levels of the HOMO and LUMO are shown in Figure 5A. The plots show that the HOMO was localized on the thio-pyrimidin ring and oxygen atom. On the other hand, the LUMO was localized on the fluoro-coumarin ring and oxygen atom. The LUMO and HOMO values of the conformer were −2.589 eV and −6.552 eV, respectively, and the energy separation between the HOMO and LUMO was observed to be ∆E = 3.962 eV. The ionization energy (I) = −EHOMO = 6.552 eV, and the electron affinity (A) = −ELUMO = 2.589 eV. The global hardness and softness can be expressed, *η* = (ELUMO−EHOMO)/2 and S = 1/2*η* of the **PC-12** compound were 1.981 eV and 0.252 eV^−1^, respectively. The softness of the molecule indicates high polarizability, more chemical reactivity, and good kinetic stability. The electrophilic index (ψ = *μ*^2^/2*η*) and electronegativity [*χ* = (I + A)/2] values were 5.273 eV and 4.571 eV, respectively [35]. The designated values of the global chemical reactivity descriptor (GCRD) parameters are presented in Supplementary Data (Table S1). As oxygen could be utilized ROS (_1_O^2^) at 0.97 eV, the compound PC-12 had a value four times higher in ROS production [36]. Furthermore, we used the B3LYP/6-311++G (d,p) level of theory to analyze the electrostatic physiochemical properties of the PC-12 molecule, and the graphical depiction is shown in Figure 5B. The resulting molecular electrostatic potential map, with a range of −5.351 × 10^−2^ to 5.351 × 10^−2^, represents its potential distribution. The oxygen (O) atom of the C=O bond of the PC-12 molecule is surrounded by an electronegative potential, representing a possible ROS-generating site.

### 2.8. PC-12 Interacts with JNK In Silico

**Figure 6:**
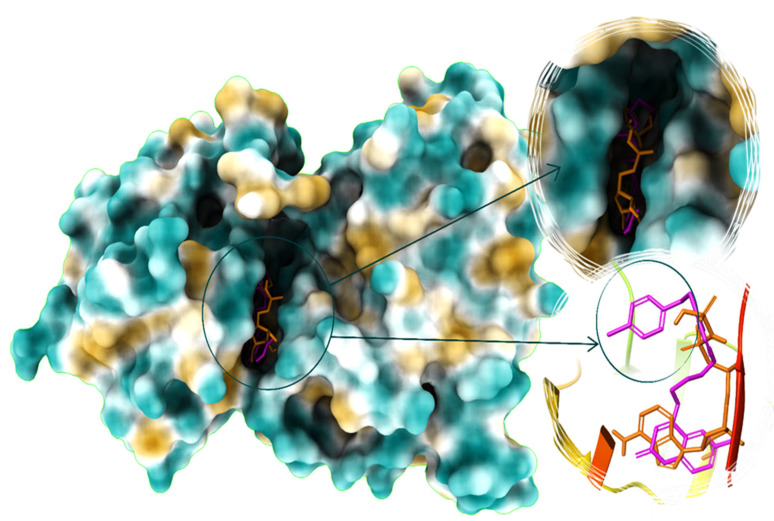
Binding pose of the **PC-12** (**magenta**) and the co-crystal ligand (**orange**) inside the binding pocket of JNK3.

Docking analysis was performed on PC-12 to evaluate its inhibitory action against the receptor. AutoDock4.2 was used to simulate the docking of PC-12 to the active site of JNK3. The docking of a co-crystallized ANP ligand to the active site of JNK3 replicated all of the critical interactions achieved by the co-crystallized ligand structure (**PDB: 1JNK**), indicating that the docking protocol was appropriate for the docking study. The binding energies of ANP and PC-12 to the active site were −6.15 and −8.8 kcal/mol, indicating that the newly synthesized compound PC-12 possessed a higher affinity for JNK3 than the co-crystallized ligand. The docking simulations revealed that the ligand had significant interactions with the receptor’s active site residues. A hydrogen bond bound the oxygen of the coumarin ring to MET149. (Figure 6).

**Figure 7:**
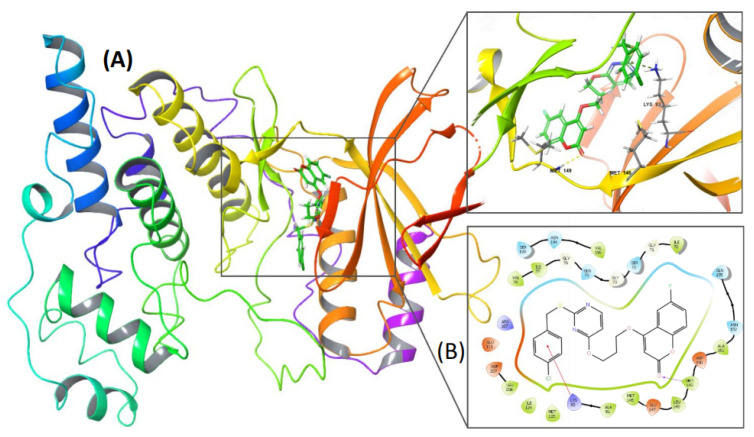
Illustrated representation of JNK3 and PC-12 complex. (**A**) Three-dimensional interaction of PC-12 with amino acid residues at the active site of the receptor. (**B**) Two-dimensional interaction diagram of JNK3 and PC-12.

Furthermore, unlike the co-crystallized ligand, the chlorobenzene group of **PC-12** entered the deep cavity. This moiety also interacted with LYS93 via pi-cation stacking, which added to the stability of PC-12 (Figure 7A,B). Hydrophobic interactions with the JNK3 active site residues ALA 91, MET115, ILE124, MET146, LEU148, MET149, and LEU206 improved the stability of the complex and, therefore, activation of JNK proceeded.

Following the docking studies, the optimum binding pose of the JNK3-PC-12 complex was subjected to molecular dynamics simulations. The molecular dynamics data provided information on the imperative stability of PC-12 in the binding pocket of the JNK3 complex during a 100 ns simulation time. Supplementary Figure S1 depicts the ligand and protein’s root mean square deviation (RMSD). The plot demonstrated that the protein RMSD gained stability at 25 ns and remained stable throughout the simulation time, implying that the protein’s stable conformation was attained after 25 ns of simulation. The ligand’s (PC-12) RMSD stabilized around 25 ns, and slight deviations were recorded for 80–90 ns. This may have been due to the change in the orientation of the chlorobenzene moiety during the simulation.

Further, the ligand retained stability until 100 ns of simulation. The root mean square fluctuation (RMSF) plot shows the protein’s N and C terminal fluctuations during the simulation time. Supplementary Figure S2 indicates that the Cα and backbone (B) factors were good, and no further deviations occurred during the 100 ns of simulation. Except for the loop portions, no fluctuations were noticed during the simulation, and the overall change was less than 2.1 Å. Overall, the results of the RMSD analyses suggest that the system was in equilibrium at the end of the simulation.

JNK3–PC-12 interaction contacts were also observed throughout the 100 ns simulation interval and are presented in the Supplementary Data (Figures S3 and S4). During 62% of the simulation period, the chlorobenzene moiety of PC-12 interacted with the LYS93 via hydrophobic and pi–cation interactions. The MET149 and ASN152 residues had 61% and 44% of the H-bond interactions, respectively. The complex’s stability was improved by the molecule’s hydrophobic connection with VAL196 (Figure S4). Overall, the interactions with the essential residues of JNK3 were consistent throughout the simulation, indicating that the docking process was valid, and that the identified complex was stable.

### 2.9. PC-12 Induced Apoptosis via Up-Regulation of JNK Pathway

**Figure 8:**
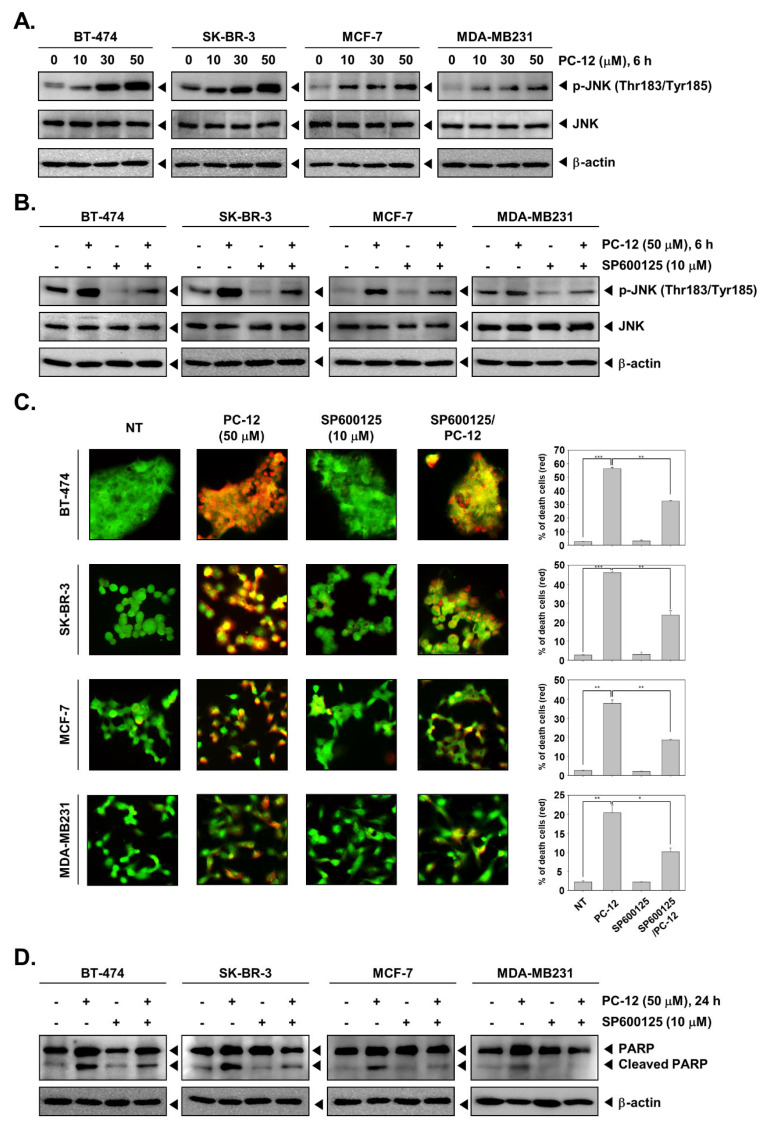
PC-12 induced PARP cleavage and cell death by activation of the JNK pathway. (**A**) The cells were treated with the indicated concentration of PC-12 for 6 h. The cells were harvested and lysed, and Western blot analysis was performed. β-ACTIN was used as input control. (**B**) The cells were treated with 0–50 µM of PC-12 or 10 µM of SP600125 for 6 h. After 6 h, the cells were collected, and Western blot analysis was performed. (**C**) The cells seeded in an 8-well chamber slide were treated with 50 µM of PC-12, 10 µM of SP600125, or a co-treatment of PC-12 (50 µM) and SP600125 (10 µM) for 24 h. After 24 h, cells were stained with calcein AM and EthD-1 for 30 min, and then live and dead cell assay was determined on a confocal microscope. *** *p* < 0.001, ** *p* < 0.01, and * *p* < 0.05 vs. PC-12-treated (NT) group. (**D**) The four cell lines were treated with the indicated concentrations of PC-12 or SP600125 for 24 h, and Western blot analysis was performed.

Next, it was determined which signaling pathway induced apoptosis upon treatment of BC cells with PC-12. One hypothesis, “breaking the brake”, states that the process of apoptosis depends on JNK expression [37]. Treatment with various concentrations of PC-12 led to increased phosphorylation of JNK (p-JNK) in all four cell lines, although JNK expression remained constant (Figure 8A). In addition, co-treatment with SP600125, a JNK inhibitor [38], reversed the effect of the increased expression of p-JNK observed with PC-12 treatment (Figure 8B). Furthermore, co-treatment of BC cells with SP600125 and PC-12 reduced the percentage of dead cells. The proportion of dead cells was reduced upon co-treatment with SP600125 and PC-12, from approximatley 20% to 10% in MDA-MB-231 cells, from 38% to 20% in MCF-7 cells, from 48% to 25% in SK-BR-3 cells, and from 55% to 30% in BT-474 cells (Figure 8C). In addition to the percentage of dead cells, the cleavage of PARP protein was also modulated. As shown in Figure 8D, PC-12 treatment promoted PARP cleavage, but the observed elevated PARP cleavage levels consequent to PC-12 treatment were reversed with SP600125 treatment. These results suggest that PC-12 induced apoptosis through increased JNK activity.

## 3. Materials and Methods

### 3.1. Experimental Materials and Method of Analysis

All chemicals and solvents were purchased from Sigma-Aldrich, Bangalore, India and TCI chemicals, Hyderabad, INDIA. Pre-coated silica gel TLC plates monitored the completion of the reaction. ^1^H and ^13^C NMR were recorded on an Agilent (400 MHz), and JEOL ECZ500R/S1 NMR spectrophotometer (500 MHz); chemical shifts are expressed as ppm. TMS and CDCl_3_ were used as internal standards and solvents. LC-MS was recorded on a Waters mass spectrometer (Xevo G2-XS QTof) (water column: Sunfire C18, 4.6 × 250 mm).

### 3.2. General Procedure for the Synthesis of Pyrimidine-Linked Coumarin Derivatives (**PC-09** to **PC-16**) (**5a–h**)

To a stirred solution of substituted 4-hydroxy coumarin (**1a,b**) (1 mmol) and anhydrous potassium carbonate (0.7 mmol) in dimethylformamide (DMF, 10 mL), 1,2-dibromoethane (1 mmol)/1-bromo-3-chloropropane (1 mmol) was added and refluxed at 70 °C for 2–3 h. Upon completion of the reaction, the precipitate was obtained with the addition of water. The residue was filtered and purified by a column chromatographic technique to yield alkylated coumarin (**2a**–**d**).

To a stirred solution of ethanol/water (1:1) containing potassium hydroxide (KOH, 1.4 mmol), 2-thiouracil (1 mmol) (**3**) was added, and the reaction mixture was heated to 45 °C with continuous stirring. Substituted benzyl bromides (1.24 mmol)/benzyl chlorides (1.24 mmol) were added to the above reaction mixture. After completion of the reaction, the solvent was distilled using a rotary evaporator, and the crude was washed with 10% aqueous NaHCO_3_ solution. The precipitate that formed was filtered and successively washed with water, ethanol, and diethyl ether to yield substituted benzyl-2-thiouracils as a white solid (**4a,b**).

Finally, the compounds (**4a,b**) (1 mmol) and (**2a**–**d**) (1 mmol) was dissolved in acetone and refluxed in the presence of potassium carbonate (2 mmol). The TLC was monitored for the reaction’s completion, and the solvent was concentrated using a rotary evaporator. The crude obtained was purified through column chromatography to yield the pyrimidine-linked coumarin derivatives (**5a**–**h**) as a white solid.

#### 3.2.1. 4-(2-((2-((4-Chlorobenzyl)thio)Pyrimidin-4-yl)oxy)Ethoxy)-2*H*-Chromen-2-One (**PC-09**) (**5a**)

White solid; MP: 128–130 °C; (153 mg) 88% yield; ^1^H NMR (400 MHz, CDCl_3_): *δ* 8.27 (d, *J* = 5.6 Hz, 1H), 7.75 (dd, *J* = 8.0, 1.2 Hz, 1H), 7.61–7.47 (m, 1H), 7.36 (d, *J* = 8.4 Hz, 2H), 7.31 (d, *J* = 8.4 Hz, 1H), 7.32–7.16 (m, 3H), 6.46 (d, *J* = 5.6 Hz, 1H), 5.69 (s, 1H), 4.79 (t, *J* = 4.4 Hz, 2H), 4.41 (t, *J* = 4.4 Hz, 2H), 4.36 (s, 2H); ^13^C NMR (100 MHz, CDCl_3_): *δ* 171.0, 168.3, 165.4, 162.7, 157.8, 153.5, 136.3, 133.1, 132.7, 130.3 (×2), 128.8 (×2), 124.1, 123.2, 116.9, 115.5, 104.4, 91.0, 67.3, 63.6, 34.7; LCMS (ESI): m/z calculated for C_22_H_17_ClN_2_O_4_S: 440.8994, found = 441.0851 [M + H]^+^ [^35^Cl], 443.0818 [M + 2H]^+^ [^37^Cl].

#### 3.2.2. 4-(3-((2-((4-Chlorobenzyl)thio)Pyrimidin-4-yl)oxy)Propoxy)-2*H*-Chromen-2-One (**PC-10**) (**5b**)

White solid; MP: 114–116 °C; (144 mg) 80% yield; ^1^H NMR (400 MHz, CDCl_3_): *δ* 8.23 (d, *J* = 5.6 Hz, 1H), 7.78 (d, *J* = 7.6 Hz, 1H), 7.60–7.46 (m, 1H), 7.39–7.18 (m, 6H), 6.40 (d, *J* = 5.6 Hz, 1H), 5.69 (s, 1H), 4.61–4.48 (m, 2H), 4.33 (s, 2H), 4.33–4.20 (m, 2H), 2.40–2.27 (m, 2H); ^13^C NMR (100 MHz, CDCl_3_): *δ* 170.8, 168.4, 165.3, 162.7, 157.4, 153.3, 136.2, 132.9, 132.4, 130.2 (×2), 128.6 (×2), 123.9, 122.9, 116.8, 115.5, 104.1, 90.7, 65.8, 62.6, 34.5, 28.1; LCMS (ESI): m/z calculated for C_23_H_19_ClN_2_O_4_S: 454.9260, found = 455.1000 [M + H]^+^ [^35^Cl], 457.0975 [M + 2H]^+^ [^37^Cl].

#### 3.2.3. 4-(2-((2-((4-Chlorobenzyl)thio)Pyrimidin-4-yl)oxy)Ethoxy)-6-Fluoro-2*H*-Chromen-2-One (**PC-11**) (**5c**)

White solid; MP: 110–112 °C; (154 mg) 85% yield; ^1^H NMR (400 MHz, CDCl_3_): *δ* 8.28 (d, *J* = 5.6 Hz, 1H), 7.47–7.35 (m, 1H), 7.36 (d, *J* = 8.0 Hz, 2H), 7.25 (d, *J* = 8.0 Hz, 4H), 6.47 (d, *J* = 5.6 Hz, 1H), 5.73 (s, 1H), 4.78 (s, 2H), 4.41 (s, 2H), 4.36 (s, 2H); ^13^C NMR (100 MHz, CDCl_3_): *δ* 170.9, 168.1, 164.3, 162.1, 159.8, 157.7, 157.4, 149.4, 136.1, 133.0, 130.1 (×2), 128.6 (×2), 120.2, 119.9 (F splits the C), 118.4, 118.3 (F splits the C), 116.4, 116.3 (F breaks the C), 109.0, 108.8 (F splits the C), 104.2, 91.6, 67.3, 63.4, 34.6; LCMS (ESI): m/z calculated for C_22_H_16_ClFN_2_O_4_S: 458.8898, found = 459.0775 [M + H]^+^ [^35^Cl], 461.0749 [M + 2H]^+^ [^37^Cl].

#### 3.2.4. 4-(3-((2-((4-Chlorobenzyl)thio)Pyrimidin-4-yl)oxy)Propoxy)-6-Fluoro-2*H*-Chromen-2-One (**PC-12**) (**5d**)

White solid; MP: 126–128 °C; (154 mg) 82% yield; ^1^H NMR (400 MHz, CDCl_3_): *δ* 8.24 (d, *J* = 5.2 Hz, 1H), 7.44 (d, *J* = 8 Hz, 1H), 7.39–7.18 (m, 6H), 6.41 (d, *J* = 5.2 Hz, 1H), 5.72 (s, 1H), 4.61–4.48 (m, 2H), 4.33 (s, 2H), 4.33–4.20 (m, 2H), 2.34 (t, *J* = 5.6 Hz, 2H); ^13^C NMR (100 MHz, CDCl_3_): *δ* 170.9, 168.3, 164.5, 162.3, 159.8, 157.4, 157.3, 149.4, 136.1, 132.9, 130.1 (×2), 128.5 (×2), 120.0, 119.8 (F splits the C), 118.4, 118.3 (F splits the C), 116.5, 116.4 (F splits the C), 108.8, 108.6 (F splits the C), 104.1, 91.4, 66.1, 62.6, 34.5, 28.0; LCMS (ESI): *m*/*z* calculated for C_23_H_18_ClFN_2_O_4_S: 472.9164, found = 473.0926 [M + H]^+^ [^35^Cl], 475.0935 [M + 2H]^+^ [^37^Cl].

#### 3.2.5. 4-(2-((2-((4-Methoxybenzyl)thio)Pyrimidin-4-yl)oxy)Ethoxy)-2*H*-Chromen-2-One(**PC-13**) (**5e**)

White solid; MP: 98–100 °C; (158 mg) 90% yield; ^1^H NMR (500 MHz, CDCl_3_): *δ* 8.29 (d, *J* = 5.5 Hz, 1H), 7.78 (dd, *J* = 8.0, 1.5 Hz, 1H), 7.62–7.49 (m, 1H), 7.35 (d, *J* = 8.5 Hz, 2H), 7.38–7.27 (m, 1H), 7.30–7.19 (m, 1H), 6.85 (d, *J* = 8.5 Hz, 2H), 6.47 (d, *J* = 5.5 Hz, 1H), 5.70 (s, 1H), 4.83 (t, *J* = 4.5 Hz, 2H), 4.41 (t, *J* = 4.5 Hz, 2H), 4.37 (s, 2H), 3.79 (s, 3H); ^13^C NMR (126 MHz, CDCl_3_): *δ* 171.4, 168.0, 165.3, 162.7, 158.7, 157.7, 153.2, 132.6, 129.9 (×2), 129.1, 124.0, 123.1, 116.7, 115.3, 113.9 (×2), 104.0, 90.7, 67.1, 63.4, 55.2, 34.8; LCMS (ESI): *m*/*z* calculated for C_23_H_20_N_2_O_5_S: 436.4803, found = 437.1395 [M + H]^+^.

#### 3.2.6. 4-(3-((2-((4-Methoxybenzyl)thio)Pyrimidin-4-yl)oxy)Propoxy)-2*H*-Chromen-2-One (**PC-14**) (**5f**)

White solid; MP: 96–98 °C; (160 mg) 88% yield; ^1^H NMR (500 MHz, CDCl_3_): *δ* 8.25 (d, *J* = 5.5 Hz, 1H), 7.86–7.74 (m, 1H), 7.62–7.48 (m, 1H), 7.39–7.26 (m, 3H), 7.31–7.19 (m, 1H), 6.83 (d, *J* = 8.5 Hz, 2H), 6.41 (d, *J* = 6.0 Hz, 1H), 5.70 (s, 1H), 4.65–4.52 (m, 2H), 4.35 (s, 2H), 4.28 (t, *J* = 6.0 Hz, 2H), 3.78 (s, 3H), 2.44–2.29 (m, 2H); ^13^C NMR (126 MHz, CDCl_3_): *δ* 171.4, 168.3, 165.4, 162.9, 158.7, 157.4, 153.2, 132.5, 130.0 (×2), 129.2, 123.9, 122.9, 116.8, 115.5, 113.8 (×2), 103.9, 90.6, 65.7, 62.5, 55.2, 34.7, 28.0; LCMS (ESI): *m*/*z* calculated for C_24_H_22_N_2_O_5_S: 450.5069, found = 451.1485 [M + H]^+^.

#### 3.2.7. 6-Fluoro-4-(2-((2-((4-Methoxybenzyl)thio)Pyrimidin-4-yl)oxy)Ethoxy)-2*H*-Chromen-2-One (**PC-15**) (**5g**)

White solid; MP: 88–90 °C; (158 mg) 86% yield; ^1^H NMR (500 MHz, CDCl_3_): *δ* 8.22 (d, *J* = 5.5 Hz, 1H), 7.44–7.32 (m, 1H), 7.28 (d, *J* = 8.5 Hz, 2H), 7.27–7.14 (m, 2H), 6.78 (d, *J* = 8.5 Hz, 2H), 6.40 (d, *J* = 5.5 Hz, 1H), 5.66 (s, 1H), 4.74 (d, *J* = 4.5 Hz, 2H), 4.33 (d, *J* = 4.5 Hz, 2H), 4.30 (s, 2H), 3.72 (s, 3H); ^13^C NMR (126 MHz, CDCl_3_): *δ* 171.4, 167.9, 164.4, 162.2, 159.5, 158.7, 157.7, 157.6, 149.3, 129.9 (×2), 129.0, 120.2, 120.0 (F splits the C), 118.4, 118.3 (F splits the C), 116.3, 116.2 (F splits the C), 113.9 (×2), 109.0, 108.8 (F splits the C), 104.0, 91.5, 67.3, 63.3, 55.2, 34.8; LCMS (ESI): *m*/*z* calculated for C_23_H_19_FN_2_O_5_S: 454.4708, found = 455.1131 [M + H]^+^.

#### 3.2.8. 6-Fluoro-4-(3-((2-((4-Methoxybenzyl)thio)Pyrimidin-4-yl)oxy)Propoxy)-2*H*-Chromen-2-One (**PC-16**) (**5h**)

White solid; MP: 130–132 °C; (159 mg) 84% yield; ^1^H NMR (500 MHz, CDCl_3_): *δ* 8.26 (d, *J* = 5.5 Hz, 1H), 7.52–7.40 (m, 1H), 7.33 (d, *J* = 9.0 Hz, 2H), 7.35–7.21 (m, 2H), 6.83 (d, *J* = 8.5 Hz, 2H), 6.42 (d, *J* = 6.0 Hz, 1H), 5.73 (s, 1H), 4.58 (t, *J* = 6.0 Hz, 2H), 4.35 (s, 2H), 4.28 (t, *J* = 6.0 Hz, 2H), 3.78 (s, 3H), 2.44–2.29 (m, 2H); ^13^C NMR (126 MHz, CDCl_3_): *δ* 171.4, 168.2, 164.5, 162.4, 159.5, 158.7, 157.6, 157.5, 149.4, 130.0 (×2), 129.1, 120.1, 119.9 (F splits the C), 118.5, 118.4 (F splits the C), 116.5, 116.4 (F splits the C), 113.8 (×2), 108.8, 108.6 (F splits the C), 103.9, 91.4, 66.1, 62.5, 55.2, 34.7, 28.0; LCMS (ESI): *m*/*z* calculated for C_24_H_21_FN_2_O_5_S: 468.4973, found = 469.1390 [M + H]^+^.

### 3.3. Reagents

The PC-12 stock solution was stored with dimethyl sulfoxide (DMSO) at −20 °C and diluted in cell culture medium before use. RPMI-1640 medium was purchased from Thermo Fisher Scientific HyClone (Waltham, MA, USA). DMSO, N-acetyl-L-cysteine (NAC), and 3-(4,5-dimethylthiazol-2-yl)-2,5-diphenyltetrazolium bromide (MTT) were obtained from Sigma (St. Louis. MO, USA). Anti-PARP, anti-cyclin D1, anti-p-JNK, and anti-JNK antibodies were purchased from Cell Signaling Technology (Massachusetts, USA). Anti-IAP-1, anti-Bcl-2, anti-survivin, anti-β-actin, and anti-p-p53 antibodies were obtained from Santa Cruz Biotechnology (Texas, Dallas, USA).

### 3.4. MTT Assay

Human breast cancer and breast epithelial cells were seeded on 96-well plates (1 × 10^4^ cells/well). These cells were incubated with 0,5,10,30,50 or 100 µM of PC-12 for 24 h. After 24 h, the MTT assay was performed. The absorbance was detected using VARIOSKAN LUX (Thermo Fisher, Waltham, MA, USA) [39,40,41,42,43].

### 3.5. Cell Cycle Analysis

The cells (5 × 10^5^ cells/well) were seeded on 35-well plates overnight. Cells were treated with PC-12 (50 µM) for 24 h, and then the cells were collected. The collected cells were fixed with EtOH overnight at 4 °C and subsequently treated with 10 µg/mL of RNase A for 1 h at 37 °C. PI (5 µL) was added for 10 min at RT and events detected using a BD Accuri^TM^ C6 Plus Flow Cytometer (BD Bioscience, New Jersey, USA) [44,45,46,47,48,49].

### 3.6. Annexin/PI Staining Assay

Human breast cancer cells were seeded on 6-well plates (5 × 10^5^ cells/well) and treated with 0–50 µM of PC-12 for 24 h. The cells were then harvested and stained with annexin/PI as described before [50].

### 3.7. Live and Dead Assay

The cells were seeded on an 8-well chamber slide (2 × 10^4^ cells/well) and treated with PC-12 (0–50 µM) for 24 h. Live, and dead staining assay was performed as described in a previous report [51].

### 3.8. Terminal Deoxynucleotidyl Transferase-Mediated dUTP Nick End Labeling (Tunel) Staining

BT-474, SK-BR-3, MCF-7, and MDA-MB-231 cells were seeded on 6-well plates (1 × 10^6^ cells/well) overnight and treated with 50 µM of PC-12 for 24 h. The cells were collected and fixed with 4% paraformaldehyde for 30 min at room temperature. After 30 min, the cells were washed with PBS and treated with 0.2% Triton x-100 for 10 min at RT. Then, the TUNEL enzyme and TUNEL label buffer were utilized for 1 h at 37 °C. The stained cells were detected and analyzed with a BD AccuriTM C6 Plus Flow Cytometer (BD Bioscience, Franklin Lakes, NJ, USA) [52].

### 3.9. Western Blot Analysis

The cells were treated with the indicated concentrations for the indicated durations, and then harvested and lysed. The samples were quantified for the same amount of proteins. Thereafter, Western blot analysis was performed as reported in a previous study [53].

### 3.10. ROS Measurement by H2DCF-DA

The cells were incubated with 50 µM PC-12 for 12 h, and then treated with 10 µM of H2DCF-DA for 30 min at 37 °C. The ROS levels were detected and analyzed using a BD AccuriTM C6 Plus Flow Cytometer (BD Bioscience, Franklin Lakes, NJ, USA).

### 3.11. GSH/GSSG Assay for ROS Detection

For measuring the GSH/GSSG ratio, GSH/GSSG-GloTM assay was used (Promega). The cells were seeded overnight on a 96-well plate (1 × 104 cells/well) and treated with 0–50 µM of PC-12 for 24 h. The assay was conducted according to the manufacturer’s protocol.

### 3.12. In Silico DFT Calculations

Computational DFT studies for the molecules were performed using Gaussian 09 [54,55,56]. The molecule was built and visualized using Gaussview 5 program package. The molecular geometry of the molecules was optimized at the B3LYP level with a 6-311++G (d, p) basis set.

### 3.13. Molecular Docking Analysis

AutoDock 4.2 [57] was used to perform a standard docking procedure for a rigid protein and a flexible ligand to investigate the binding pose of PC-12 in the binding pocket of the JNK3 inhibitor. The X-ray crystallographic structure of JNK3 (PDB: 1JNK) was retrieved from the Protein Data Bank and used for molecular docking analysis. The protein was prepared before the docking simulations by removing the crystalline water, adding polar hydrogen atoms, and updating the missing residues. In the crystal structure, a grid box (80 Å × 80 Å × 80 Å) with a spacing of 0.375 Å was created and centered on the ligand’s mass center. Before the docking, Autogrid 4 was used to generate energy grid maps for all possible ligand atom types. The original ligand (ANP) was initially docked to the binding pocket and demonstrated the docking method’s plausibility. Later, ANP and **PC-12** were docked exclusively to the active site of JNK3. Finally, the docking modes were investigated using Maestro (Schrödinger Inc., New York, NY, USA) [58] and ChimerX software version 1.0 [59].

### 3.14. Molecular Dynamics Simulations

Desmond v3.8 molecular dynamics computations were used to investigate the stability of the JNK3-PC-12 complex. Using the OPLS 2005 force field, the energy of the docked complex was minimized. This was included in the water model of the simple point charge (TIP3P) in the orthorhombic box with dimensions of 10 Å × 10 Å × 10 Å. After being neutralized with counter ions, the physiological salt content remained constant at 0.15 M. The simulation protocol specified the system using periodic boundary conditions, the particle mesh Ewald (PME) approach for electrostatics, a 10 Å cutoff for Lennard–Jones interactions, and the SHAKE algorithm. The program also restricted the mobility of all covalent bonds containing hydrogen atoms. The ligand–receptor complex was minimized and simulated for 100 ns without constraints using an NPT ensemble (temperature of 300 K and pressure of 1.01325 bars). During the early simulations, Berendsen thermostats and barostats were employed to control the temperatures and pressures. MD simulations were run for 100 ns to analyze the docked complex’s trajectory, root means square deviation (RMSD), root mean square fluctuations (RMSF), and inter-molecular hydrogen bond interactions.

### 3.15. Statistical Analysis

All values are represented as the mean ± SD. Student’s unpaired t-test was conducted for statistical significance. *** *p* < 0.001, ** *p* < 0.01, and * *p* < 0.05 vs. non-treated (NT) cells.

## 4. Discussion

The mitogen-activated protein kinase (MAPK) family member, JNK, has been reported to be associated with a variety of cellular processes, including apoptosis, inflammation, and the stress response [60]. In addition, JNK is activated by various stimuli, such as cytokines, growth factors, and environmental stressors. One of the ways JNK can be activated is through phosphorylation, either by drug treatment, which includes anisomycin, paclitaxel, doxorubicin, sorbitol, sorafenib, resveratrol, cisplatin, or by UV irradiation [61]. Similarly, some drugs used to treat inflammation, such as nonsteroidal anti-inflammatory drugs (NSAIDs), can also increase JNK phosphorylation as part of their mechanism of action. Mainly, these drugs induce JNK phosphorylation at the Thr-Pro-Tyr residues, leading to downstream effects on cellular processes, such as apoptosis and inflammation [62].

Studies have shown ROS can induce JNK phosphorylation in human breast cancer cells. ROS-induced JNK phosphorylation has been linked to the activation of pro-apoptotic pathways and the inhibition of cell proliferation, ultimately resulting in cell death and a reduction in cancer growth. The mechanism by which ROS induces JNK phosphorylation is thought to involve the activation of upstream kinases, such as MAP kinase kinase (MKK) 4 and MKK7, which phosphorylate and activate JNK. ROS may activate these kinases through various mechanisms, including the oxidation of critical cysteine residues in these proteins [63]. The ability of ROS to promote JNK phosphorylation in breast cancer cells has important implications for cancer treatment and prevention.

In this report, it was demonstrated that the newly designed compound PC-12 induced apoptosis in BC cells by ROS generation and the activation of JNK. The multikinase inhibitor sorafenib also activates the upstream kinase MKK4 and subsequently leads to JNK phosphorylation; and which is used to treat liver, kidney, and thyroid cancer [64]. A chemotherapeutic drug, paclitaxel, which stabilizes microtubules, also results in the phosphorylation of JNK. Cisplatin has also been shown to promotes JNK phosphorylation in ovarian cancer cells, leading to apoptosis and tumor growth inhibition [65]. Furthermore, doxorubicin induces oxidative stress and ROS generation, leading to JNK phosphorylation in various types of cancer. In addition, anisomycin is an antibiotic that induces JNK phosphorylation in different types of cells, including cancer cells, leading to pro-apoptotic effects. The natural compound resveratrol also induces JNK phosphorylation in various cell types, including cancer cells, and triggers proapoptotic signaling.

Overall, the induction of JNK phosphorylation by NCEs [66,67,68,69], such as PC-12, could be a new therapeutic strategy for inhibition of cancer cell proliferation and survival. However, a detailed study is also warranted to understand the impact of medications on JNK modulation and determine the possible side effects of JNK-targeted treatments.

## 5. Conclusions

Structure-based design and synthesis of JNK inhibitors was achieved and which were observed to selectively inhibit the proliferation and survival of HER2-positive BC cells. Furthermore, in vitro, in silico, and theoretical calculations confirmed that environmentally available coumarin- and pyrimidine-based organic small molecules interact with JNK. In conclusion, a novel promoter of JNK phosphorylation, which inhibits the proliferation and survival of HER2-positive BC cells via ROS/JNK-related pathways has been identified. This novel inducer of INK phosphorylation may be of utility for mechanistic studies in BC cells.

## Figures and Tables

**Table 1:** Structural, physical, and cell viability data of compounds (**5a**–**h**).

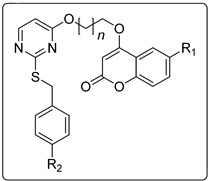				
**Compound Code**	**R_1_**	**R_2_**	***n***	**MCF-7** **(IC_50_ in μM)**
**PC-09** (**5a**)	H	Cl	1	31.29
**PC-10** (**5b**)	H	Cl	2	16.42
**PC-11** (**5c**)	F	Cl	1	47.22
**PC-12** (**5d**)	F	Cl	2	8.00
**PC-13** (**5e**)	H	OCH_3_	1	>100
**PC-14** (**5f**)	H	OCH_3_	2	13.33
**PC-15** (**5g**)	F	OCH_3_	1	>100
**PC-16** (**5h**)	F	OCH_3_	2	>100

## Data Availability

Data will be made available upon request. Supplementary Data are available online.

## Supplementary Materials

The following supporting information can be downloaded at: https://www.mdpi.com/article/10.3390/molecules28083450/s1, Supplementary Data containing data on chemical synthesis, and cytotoxicity.

## Sample Availability

Samples of the compounds are available from Dr. Basappa.

## Abbreviations

HER2: Human epidermal growth factor receptor 2. MTT assay: (3-(4,5-dimethylthiazol-2-yl)-2,5-diphenyltetrazolium bromide) tetrazolium reduction assay. ROS: Reactive oxygen species. DMSO: Dimethyl sulfoxide.
