# Supplementary material for: Discovery of Pyrimidine- and Coumarin-Linked Hybrid Molecules as Inducers of JNK Phosphorylation through ROS Generation in Breast Cancer Cells
Source: Molecules. 2023 Apr 13;28(8):3450. doi: 10.3390/molecules28083450 (PMC10142175; doi:10.3390/molecules28083450)
Supplement: Supplementary file 1 [file molecules-28-03450-s001.zip › molecules-2318141-supplementary.pdf]

Supporting information

Table S1: FMOs energy with global chemical parameters values of **PC-12** compound.

Global Parameters	PC-12
E_{HOMO} (eV)	-6.552
E_{LUMO} (eV)	-2.589
$\Delta E_{LUMO-HOMO}$ (eV)	3.962
Ionization potential (I) (eV)	6.552
Electron affinity (A) (eV)	2.589
Hardness (η) (eV)	1.981
Softness (S) $(eV)^{-1}$	0.252
Chemical potential (μ) (eV)	-4.571
Electronegativity (χ) (eV)	4.571
Electrophilicity (ψ) (eV)	5.273

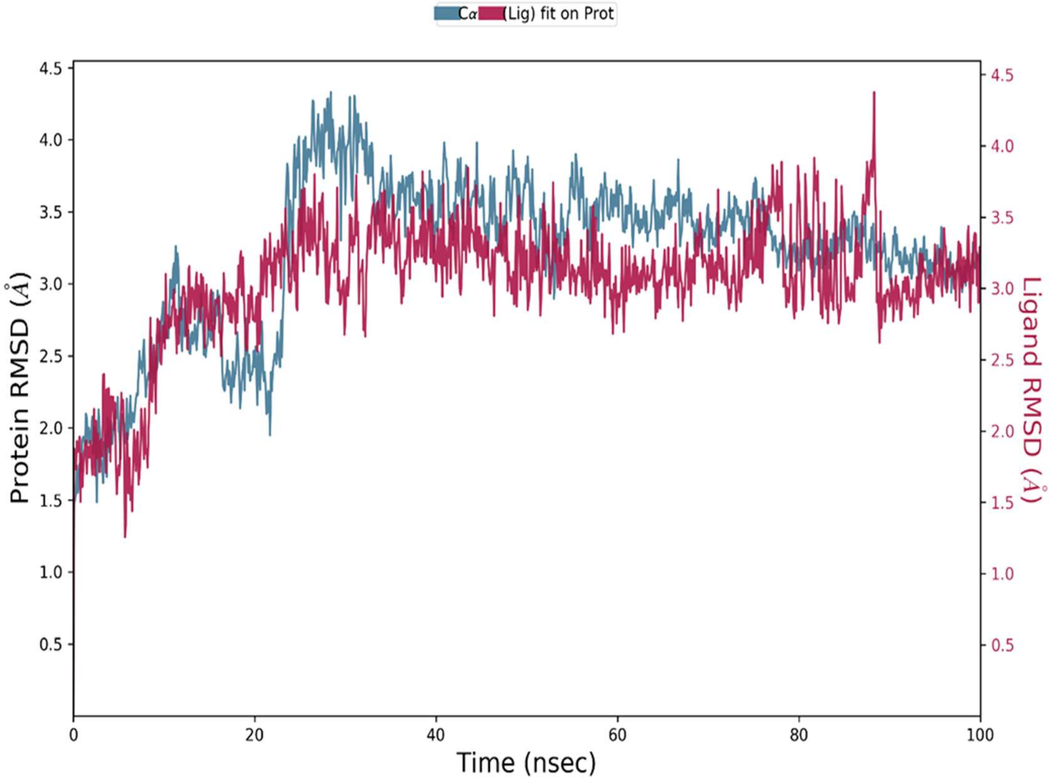

Figure S1. RMSD of **JNK3-PC-12** complex for 100 ns of MD simulations.

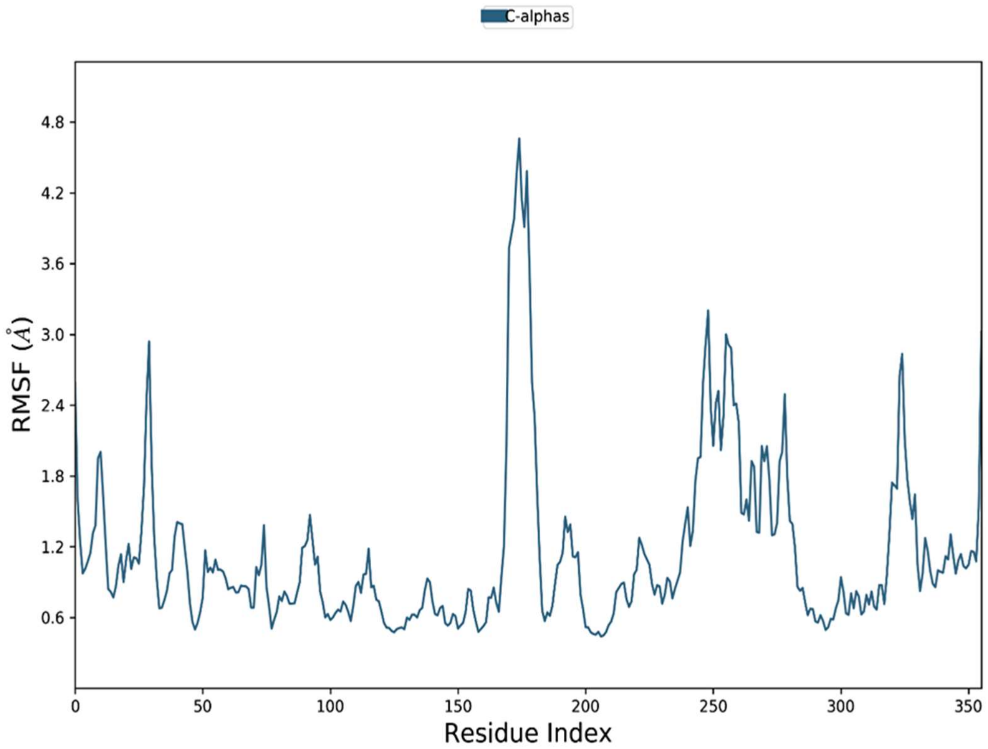

Figure S2. RMSF plot residues of JNK3 during the 100 ns simulation time

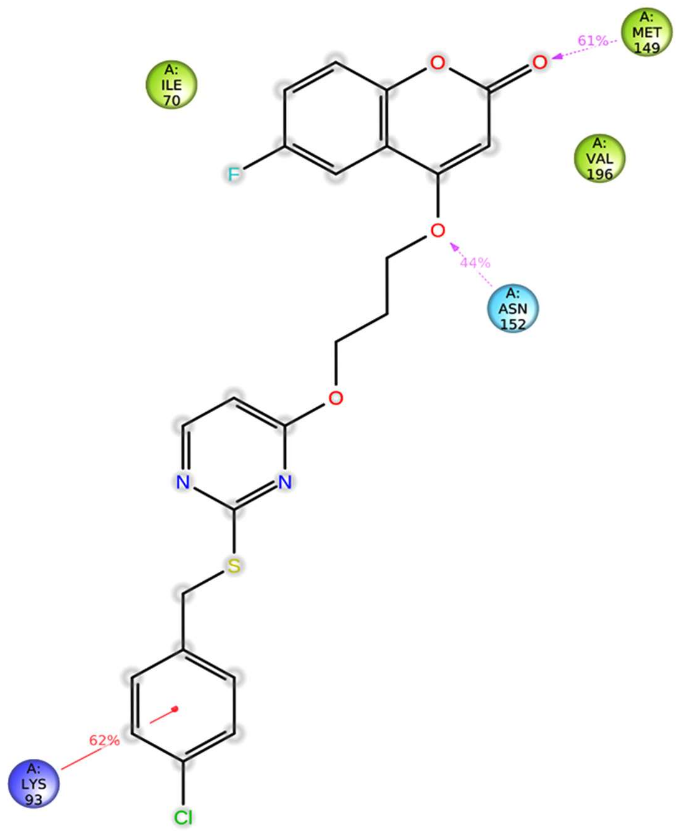

Figure S3. Simulation interaction diagram of **JNK3-PC-12** during MD simulations.

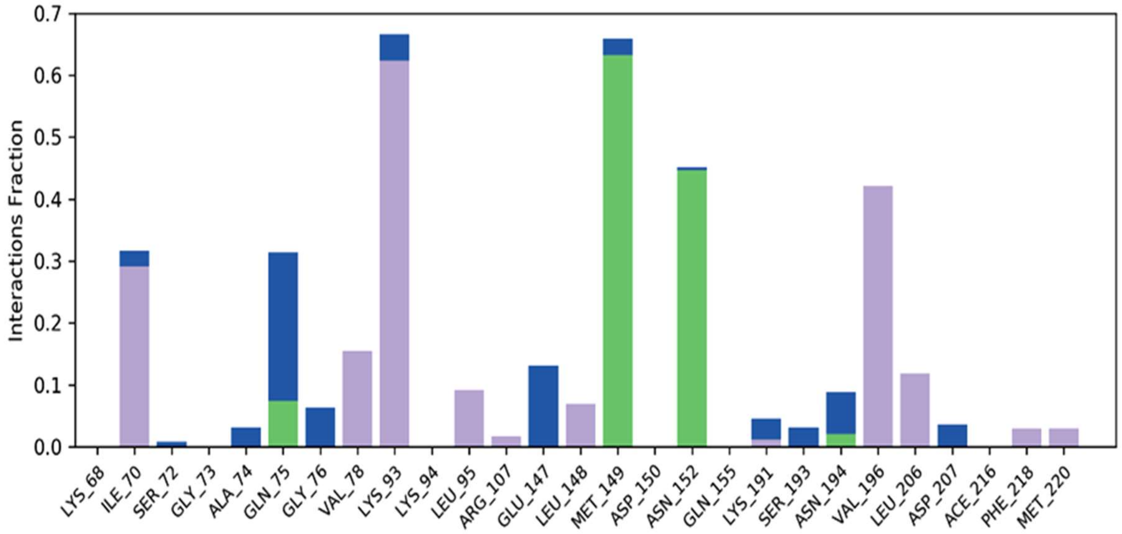

Figure S4. Bar graph showing the interaction, contact, and fraction folds of **PC-12** with residues of JNK3.

^{1}H, ^{13}C NMR, LC-MS, and log curve of the synthesized compounds

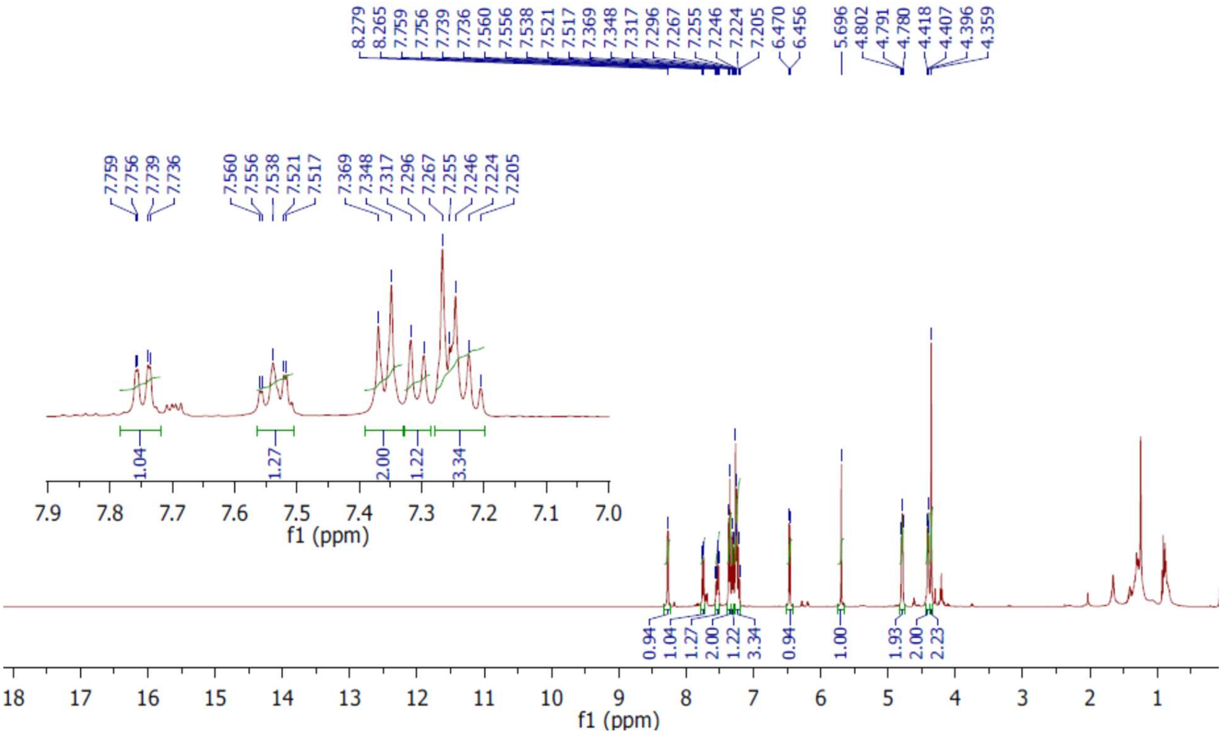

^{1}H NMR spectrum of PC-09 (5a)

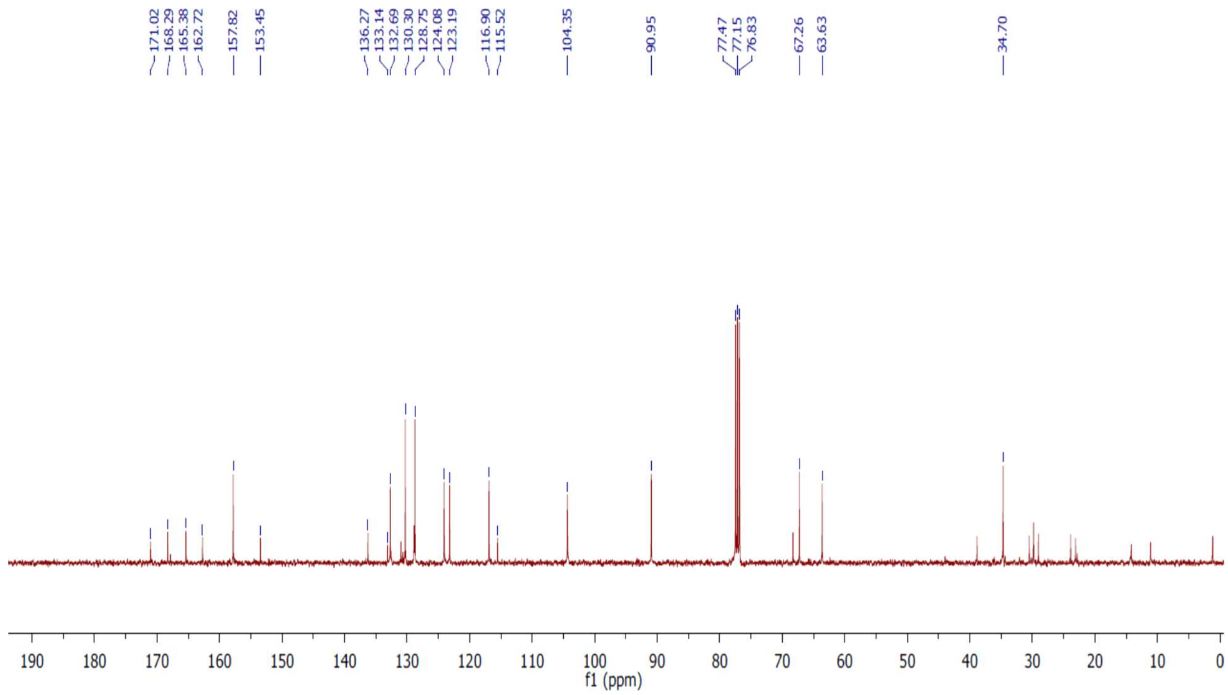

^{13}C NMR spectrum of PC-09 (5a)

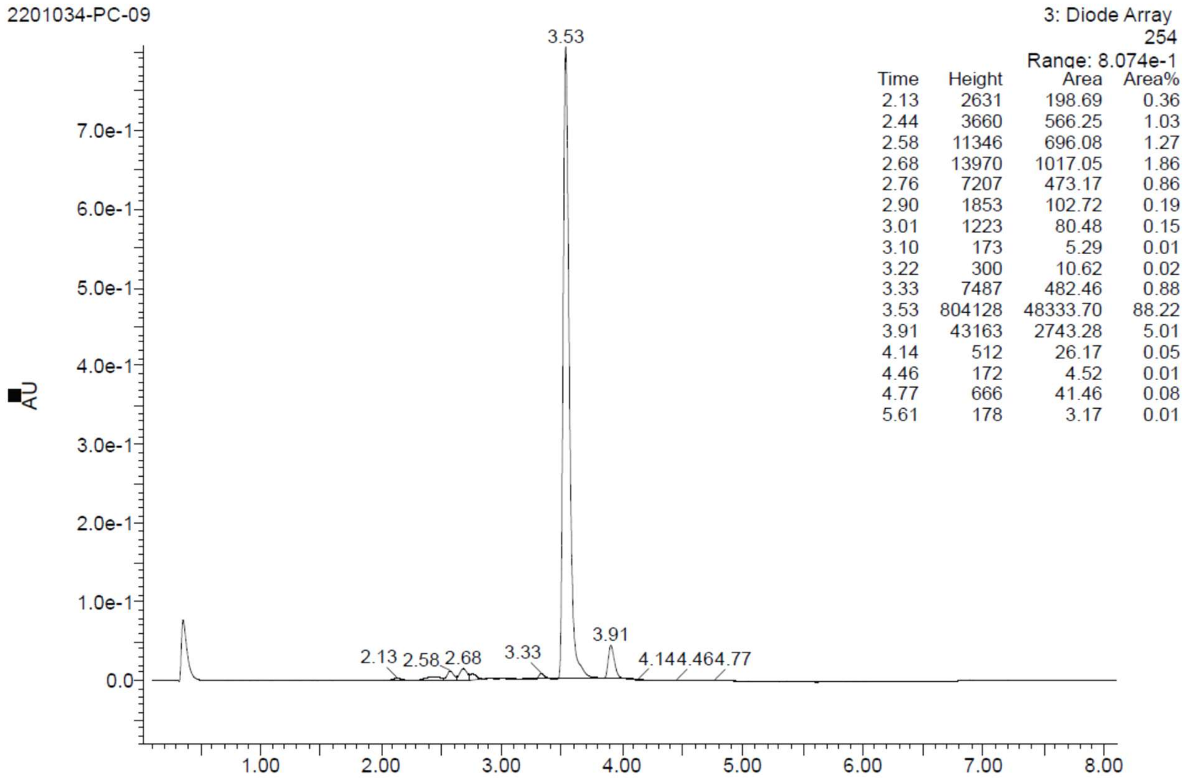

LC of PC-09 (5a)

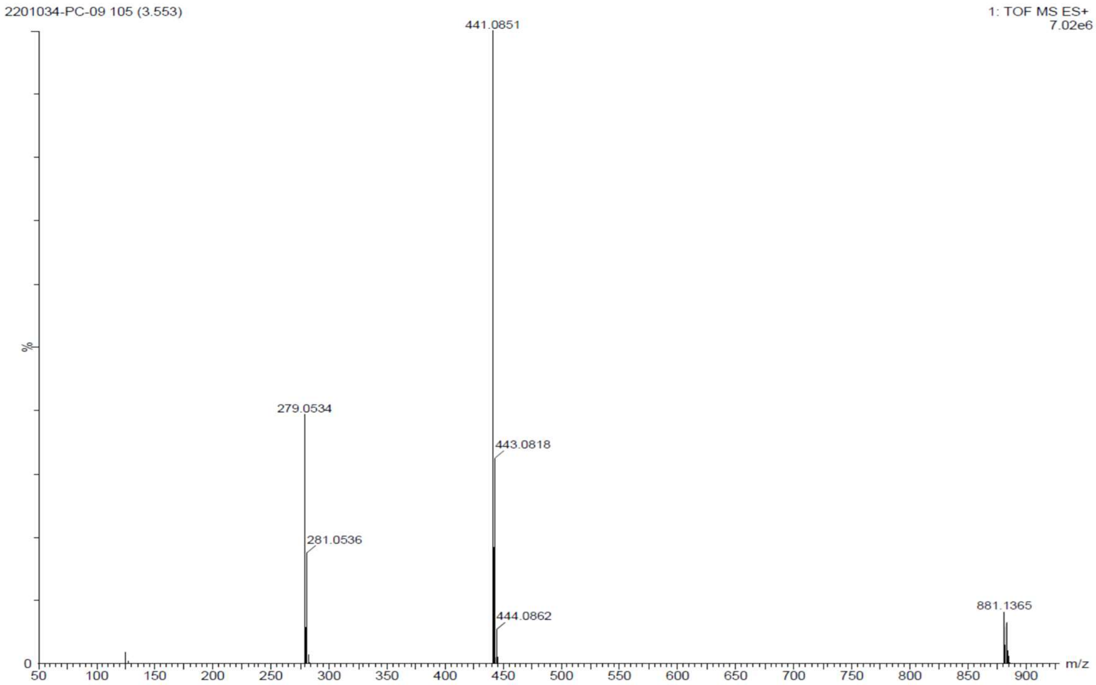

Mass spectrum of PC-09 (5a)

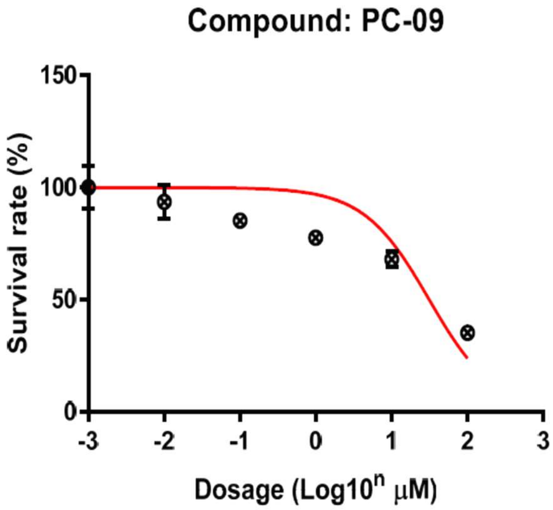

IC_{50} (μM) = 31.29
Log IC_{50} (μM) = 1.50

- Cell line: MCF7 (2000 cells/per well[96])
- Treated time: 72hrs
- Assay: MTT (90mins incubated)
- Data: PC-09

Conc. (μM)	Viability	
	AVE.	± SD.
0	100.00	6.79
0.01	93.57	7.58
0.1	85.23	1.80
1	77.56	1.57
10	67.92	3.53
100	35.25	0.26

Log curve of PC-09 (5a)

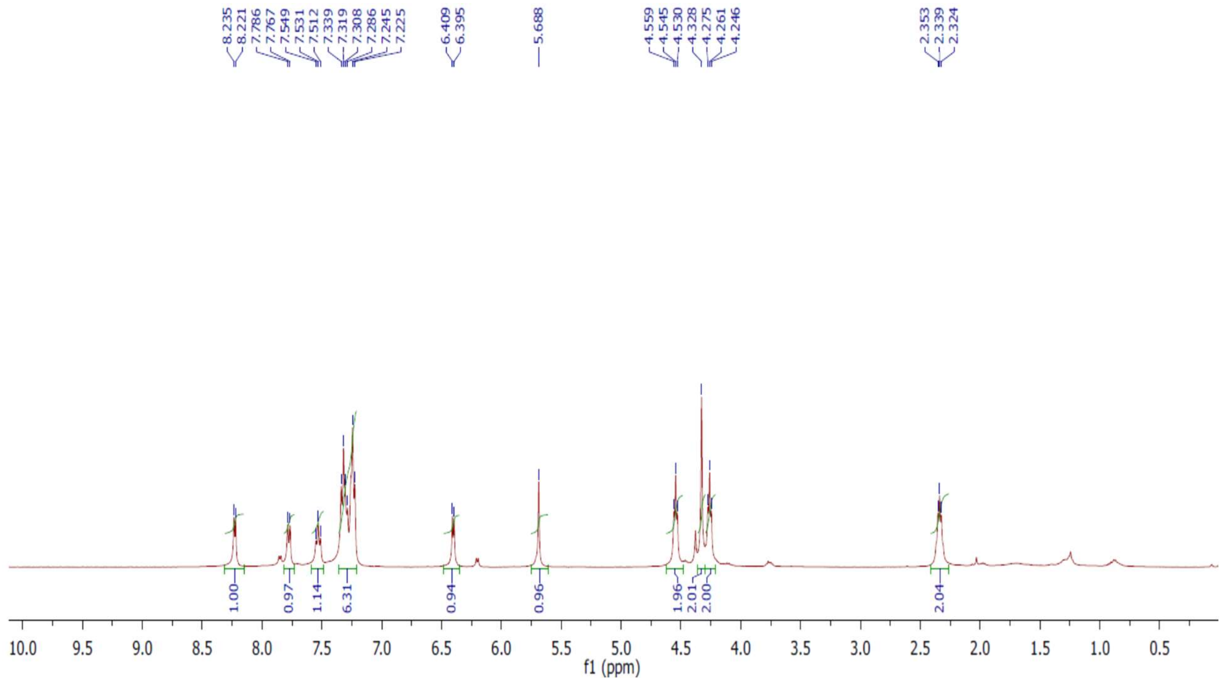

^{1}H NMR spectrum of PC-10 (5b)

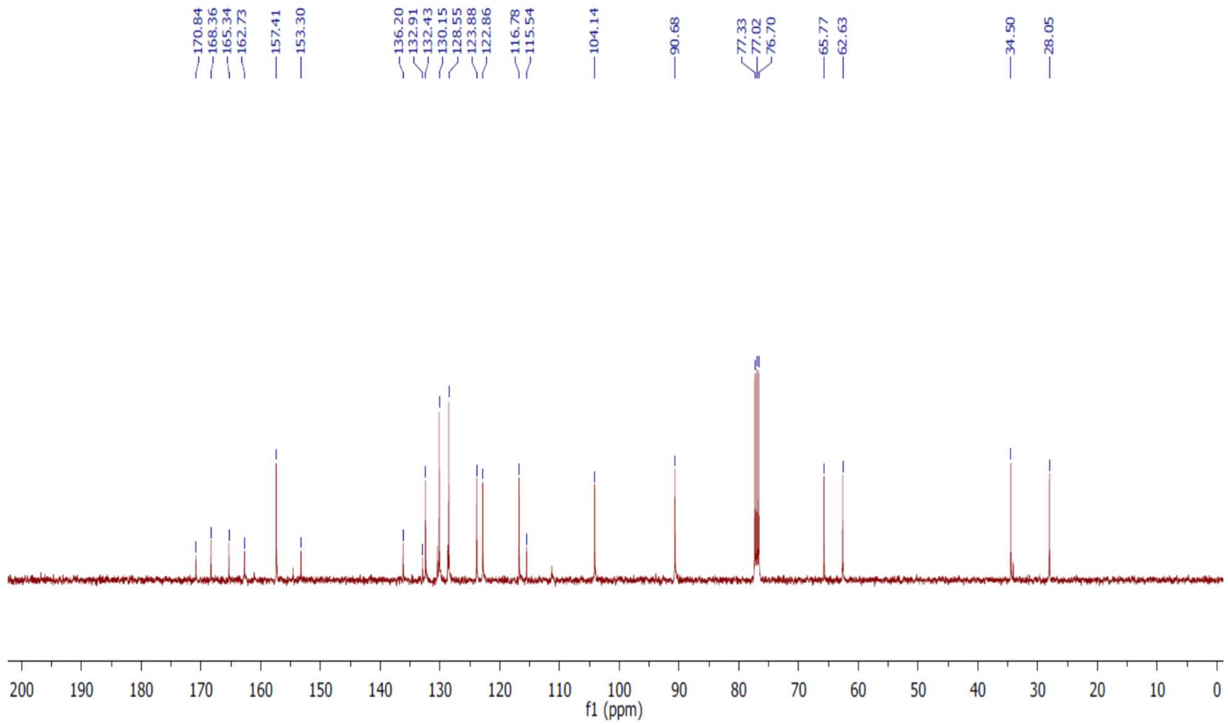

^{13}C NMR spectrum of PC-10 (5b)

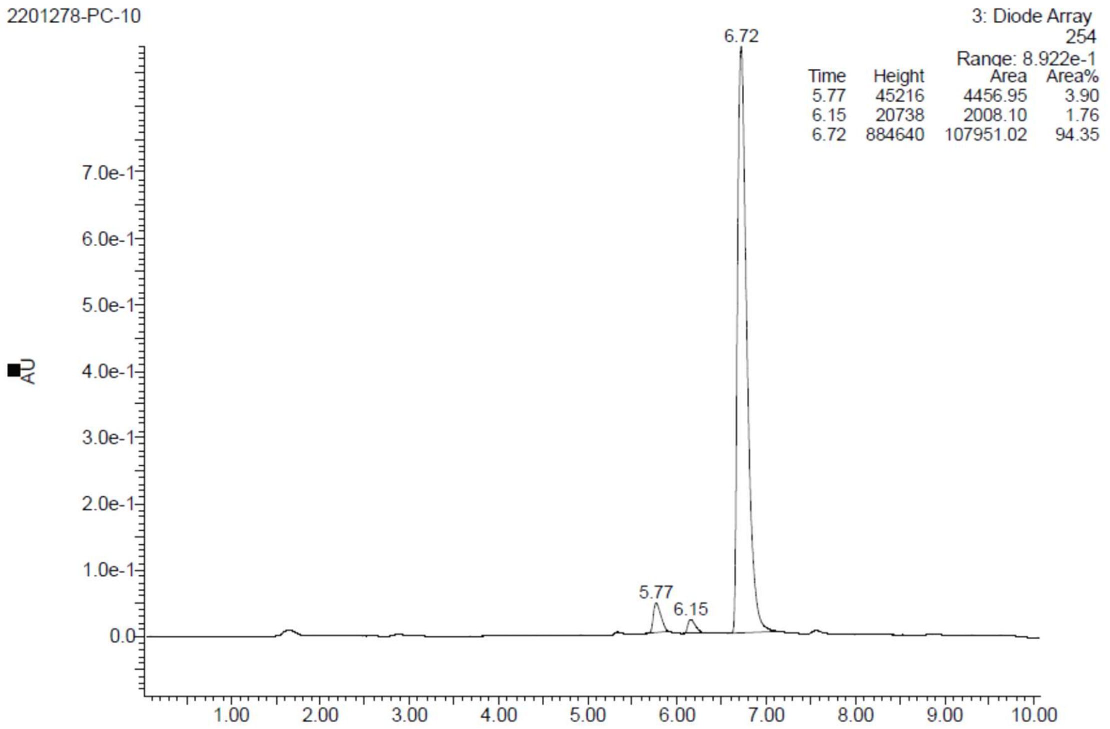

LC of PC-10 (5b)

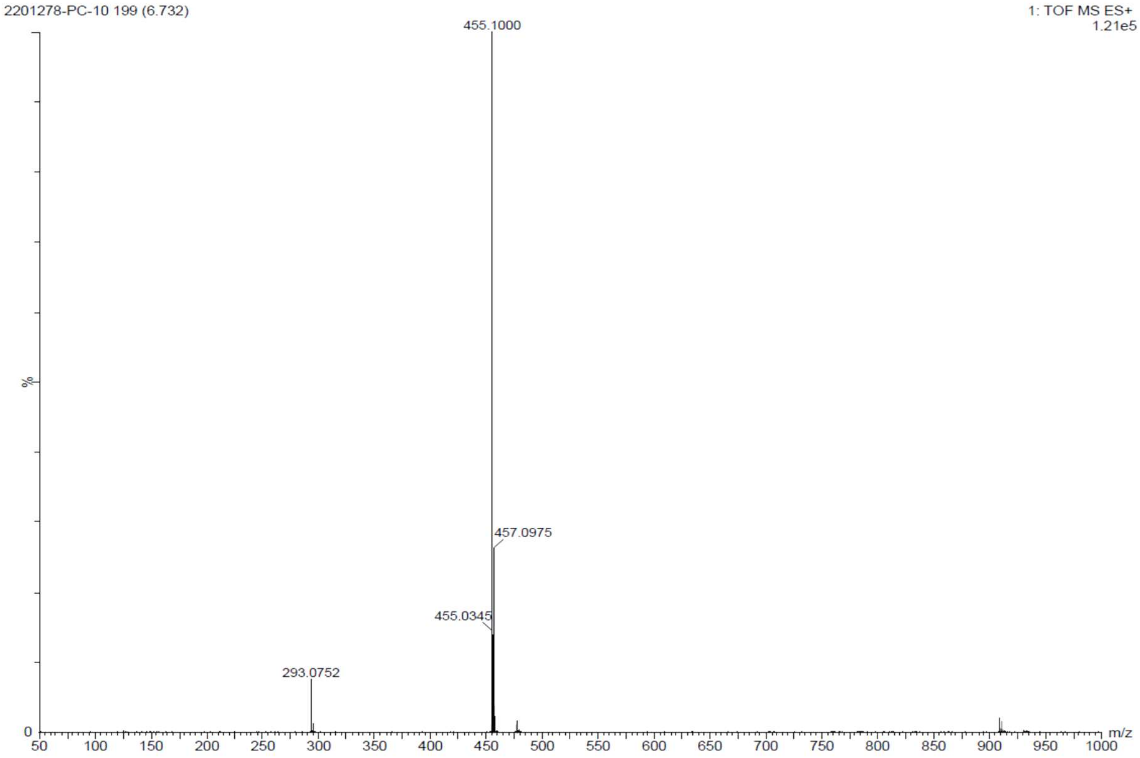

Mass spectrum of PC-10 (5b)

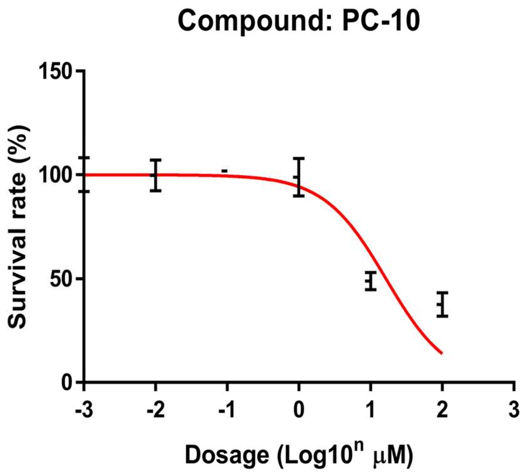

- Cell line: MCF7 (2000 cells/per well[96])
- Treated time: 72hrs
- Assay: MTT (90mins incubated)
- Data: PC-10

Conc. (μM)	Viability	
	AVE.	± SD.
**0**	100.00	8.17
**0.01**	99.70	7.40
**0.1**	101.85	2.60
**1**	98.78	9.04
**10**	48.93	4.08
**100**	37.64	5.63

IC_{50} (μM) = 16.42
Log IC_{50} (μM) = 1.22

Log curve of PC-10 (5b)

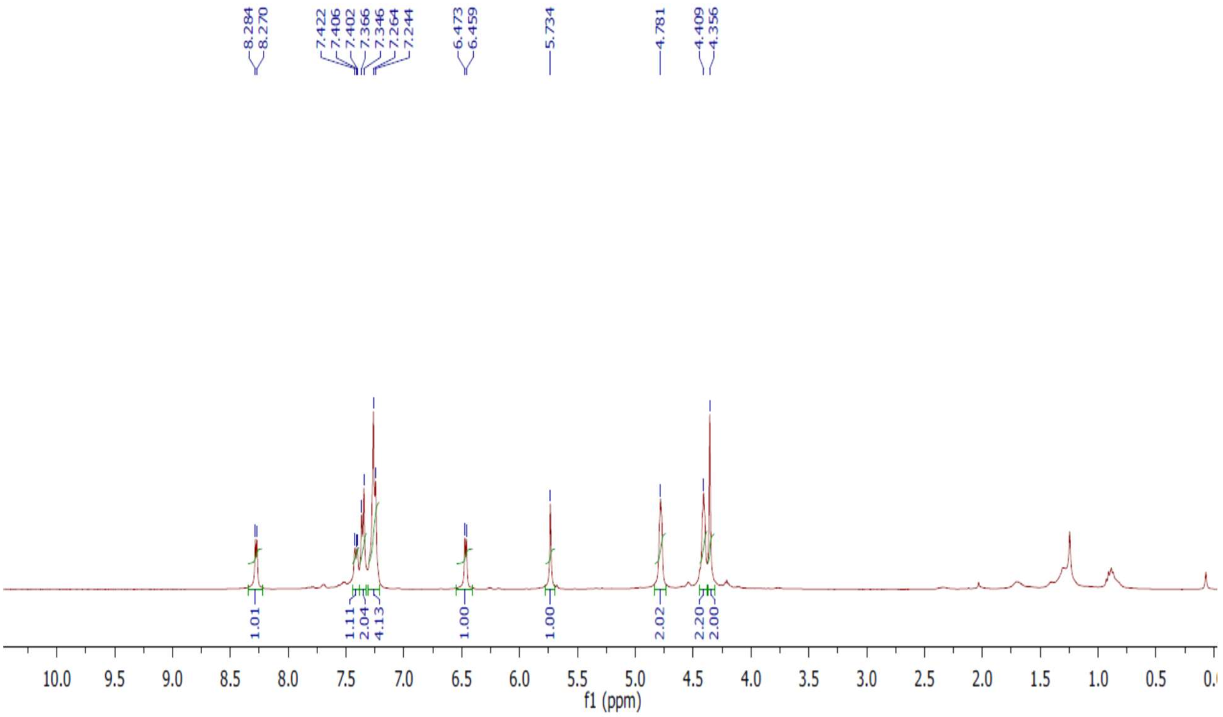

^{1}H NMR spectrum of PC-11 (5c)

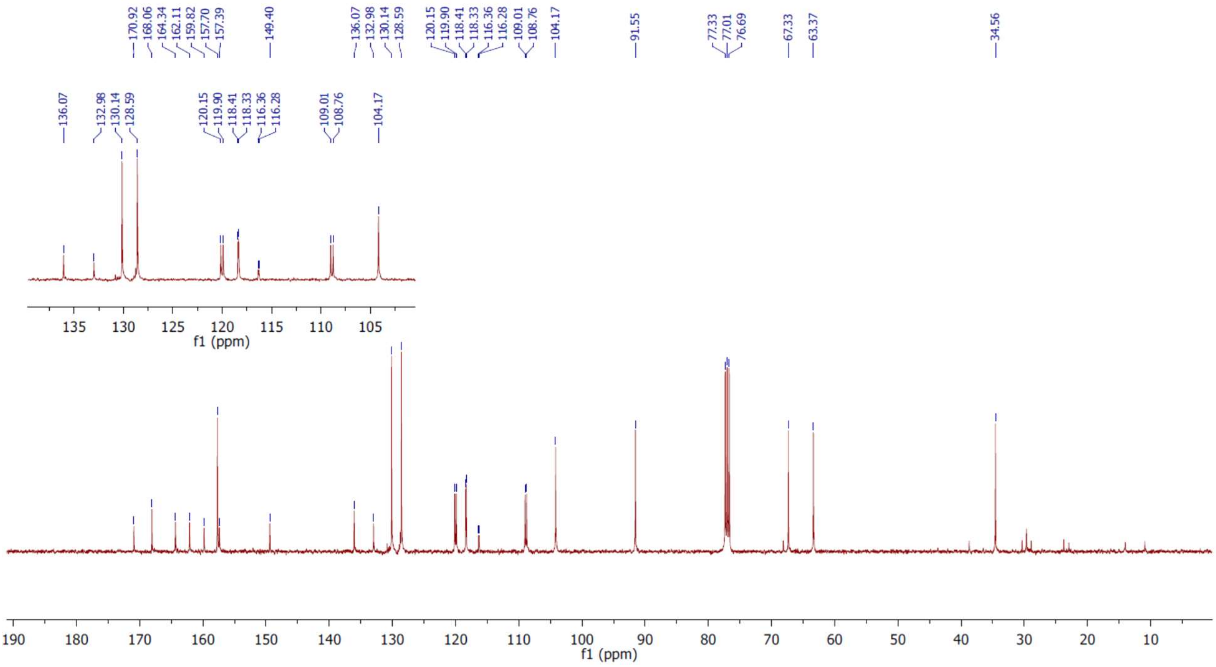

^{13}C NMR spectrum of PC-11 (5c)

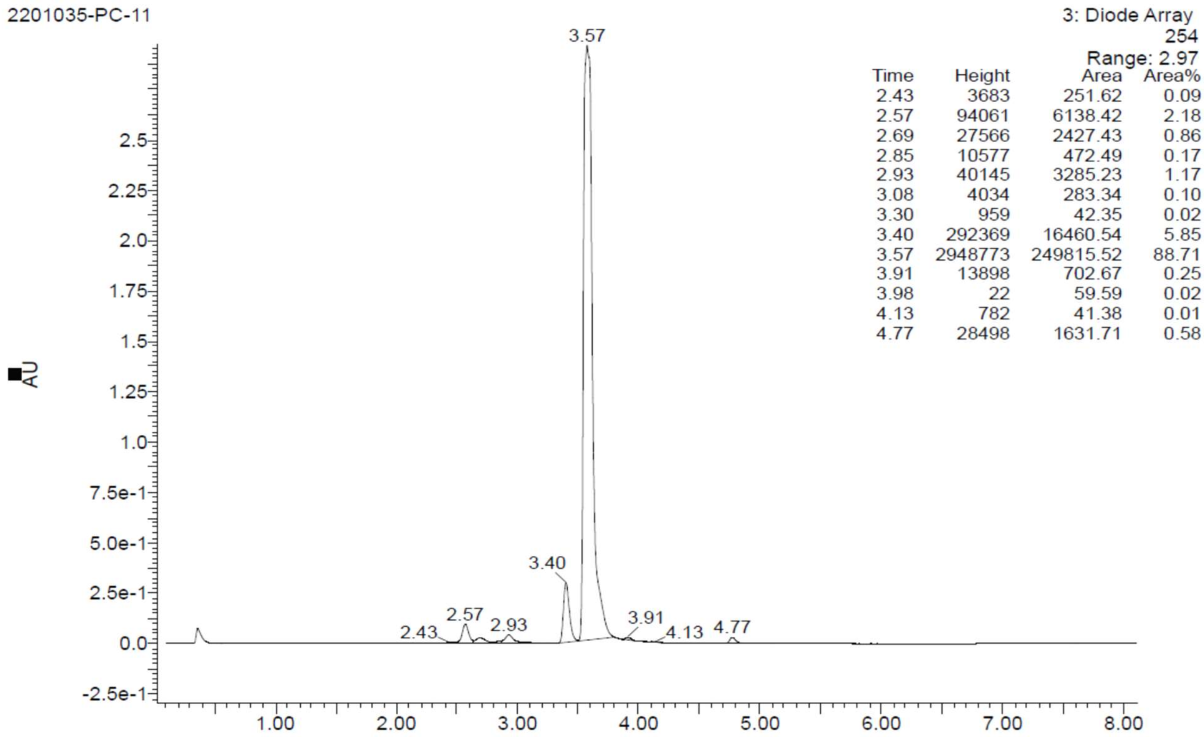

LC of PC-11 (5c)

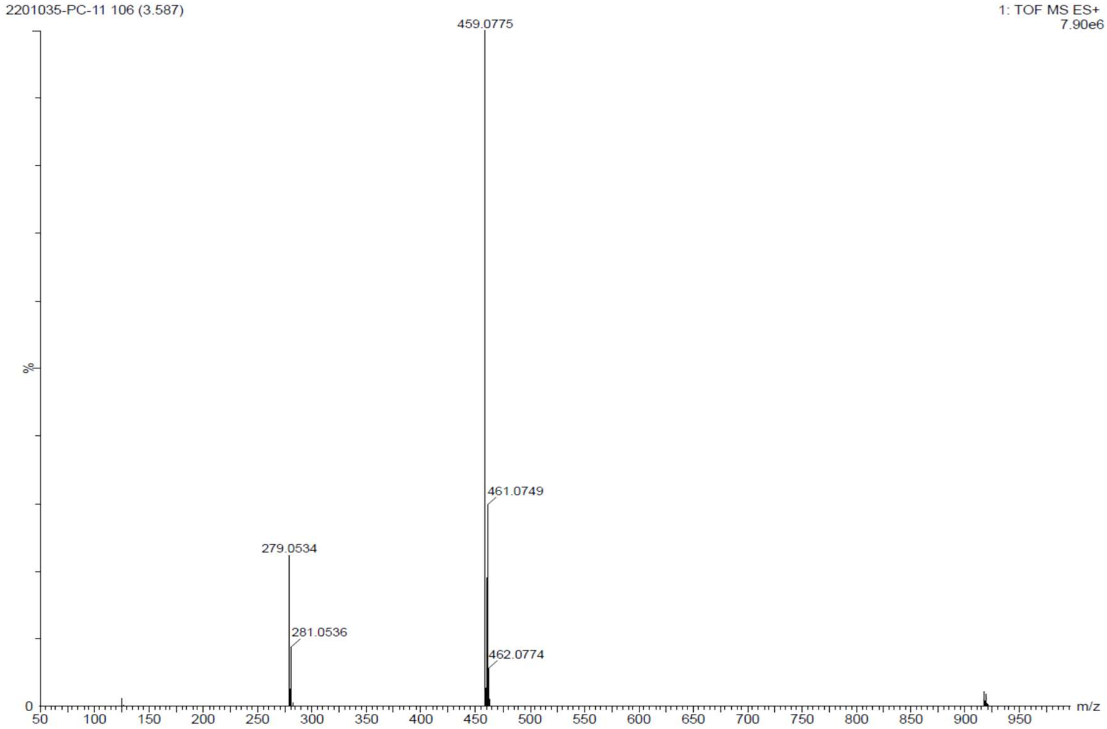

Mass spectrum of PC-11 (5c)

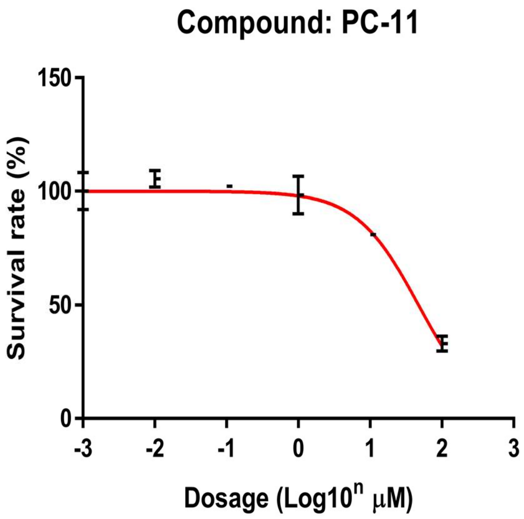

- Cell line: MCF7 (2000 cells/per well[96])
- Treated time: 72hrs
- Assay: MTT (90mins incubated)
- Data: PC-11

Conc. (μM)	Viability	
	AVE.	± SD.
**0**	100.00	8.17
**0.01**	105.41	3.67
**0.1**	102.25	2.96
**1**	98.30	8.25
**10**	80.95	0.98
**100**	33.05	3.23

Log curve of PC-11 (5c)

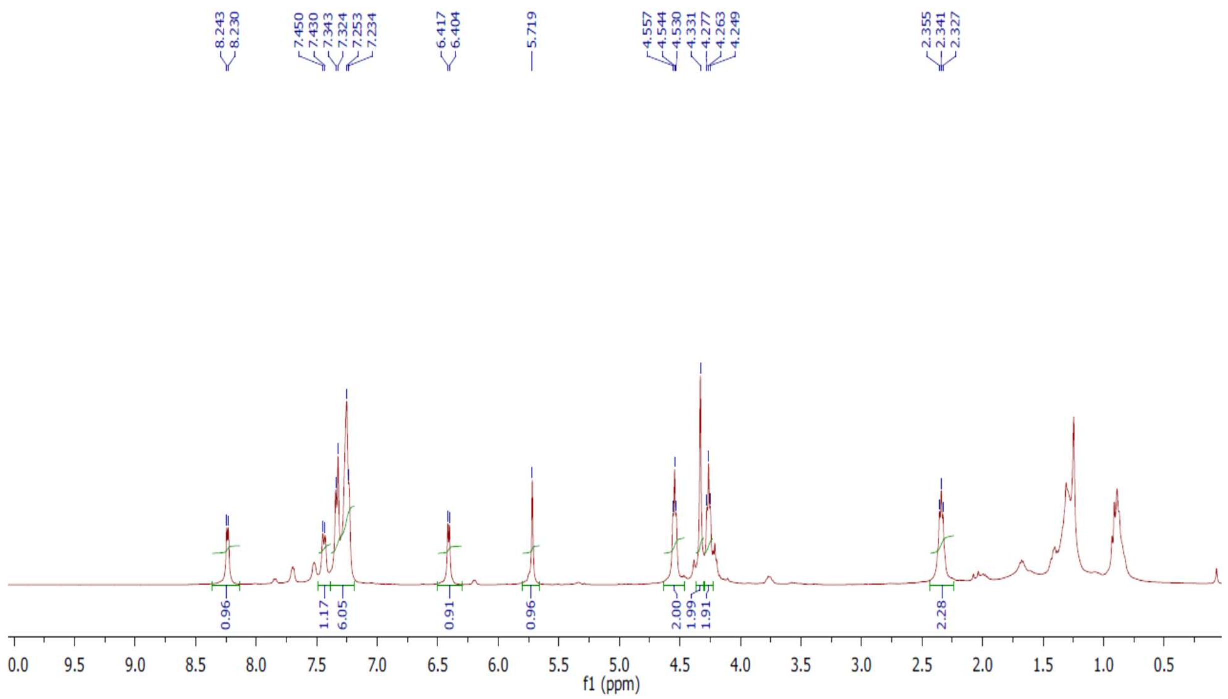

^{1}H NMR spectrum of PC-12 (5d)

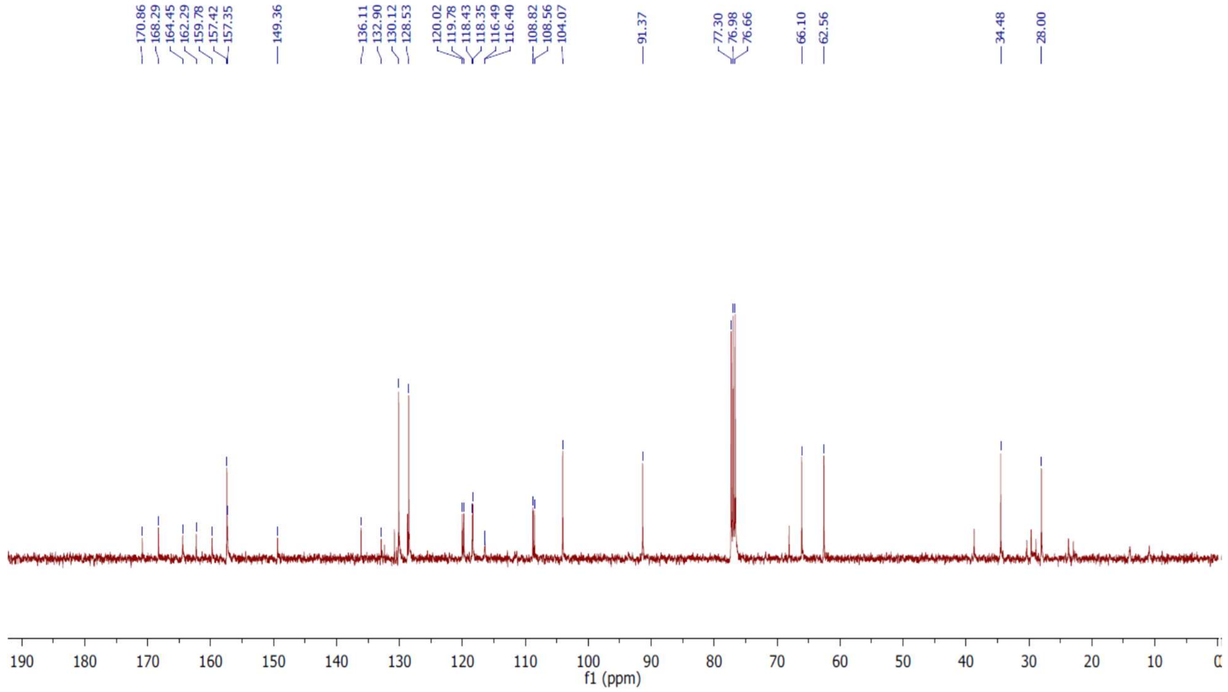

^{13}C NMR spectrum of PC-12 (5d)

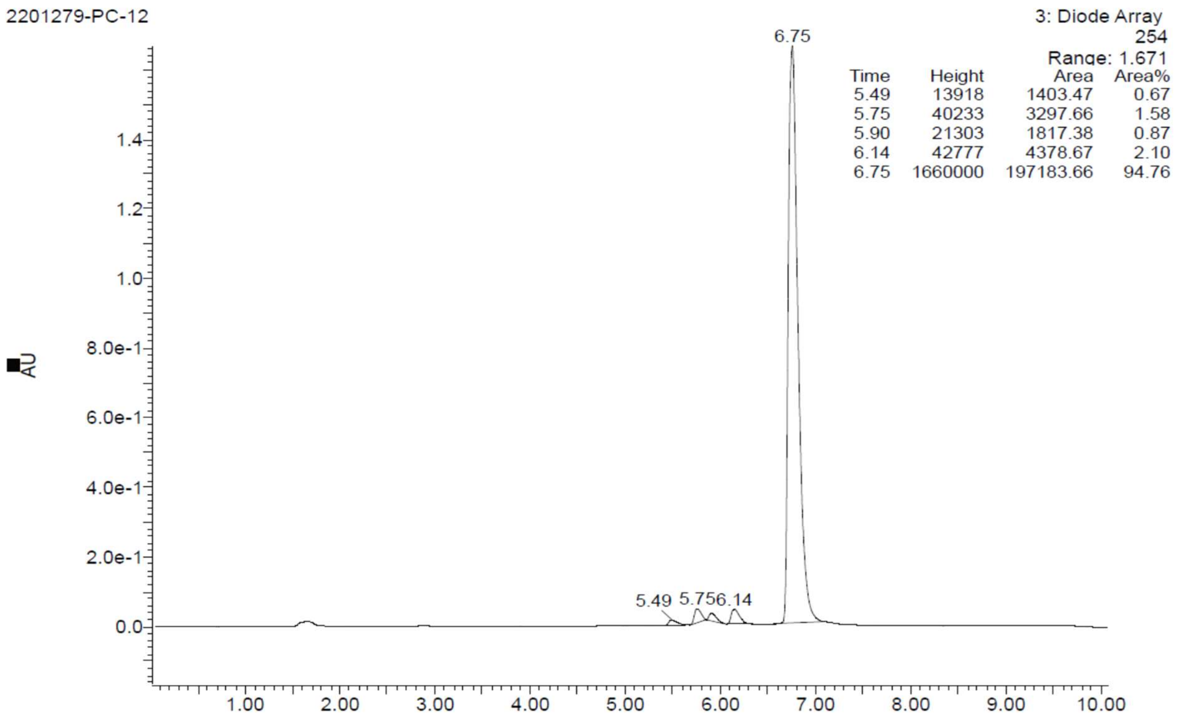

LC of PC-12 (5d)

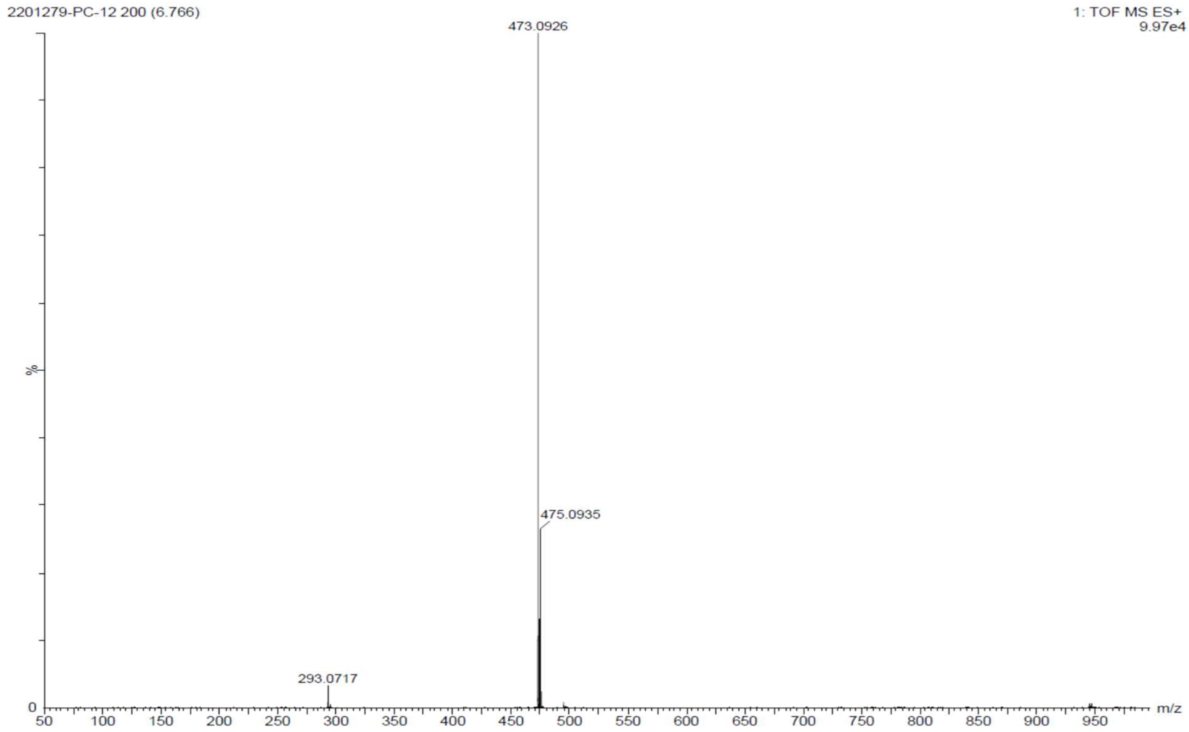

Mass spectrum of PC-12 (5d)

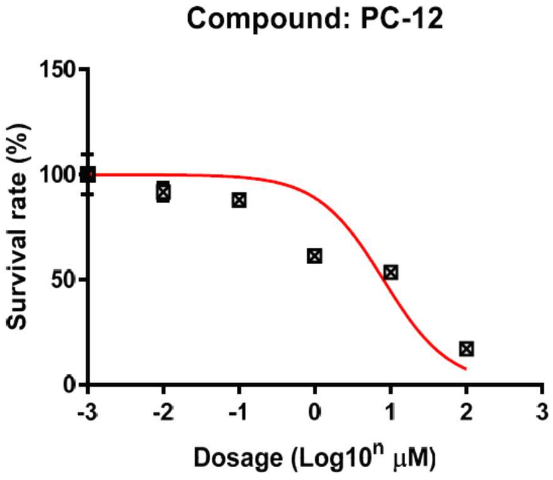

IC_{50} (μM) = 8.00
Log IC_{50} (μM) = 0.90

- Cell line: MCF7 (2000 cells/per well[96])
- Treated time: 72hrs
- Assay: MTT (90mins incubated)
- Data: PC-12

Conc. (μM)	Viability	
	AVE.	± SD.
0	100.00	6.79
0.01	91.66	4.26
0.1	87.87	2.72
1	61.31	1.42
10	53.39	1.48
100	17.02	0.54

Log curve of PC-12 (5d)

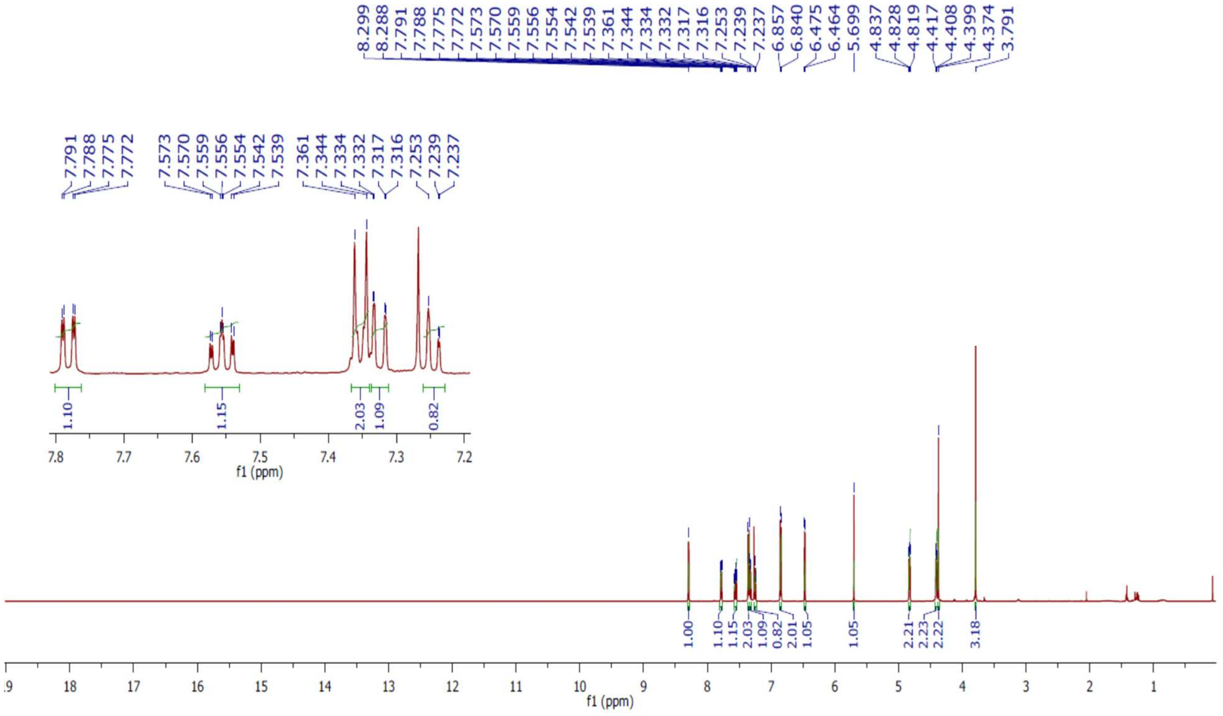

^{1}H NMR spectrum of PC-13 (5e)

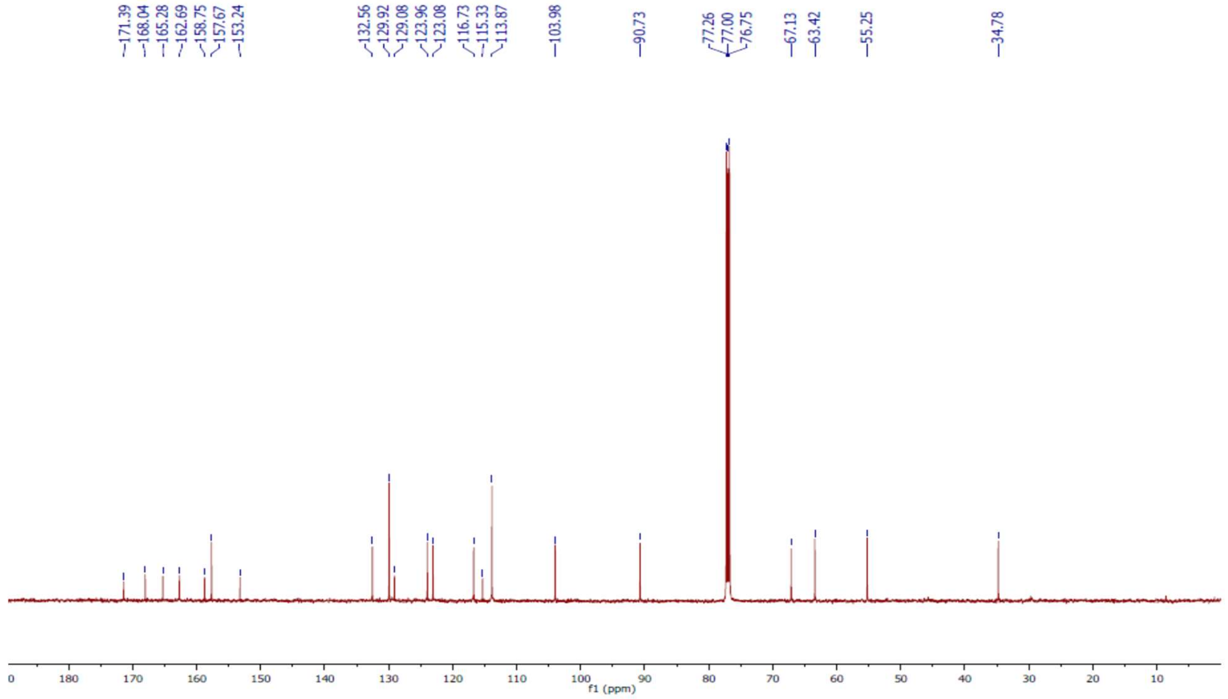

^{13}C NMR spectrum of PC-13 (5e)

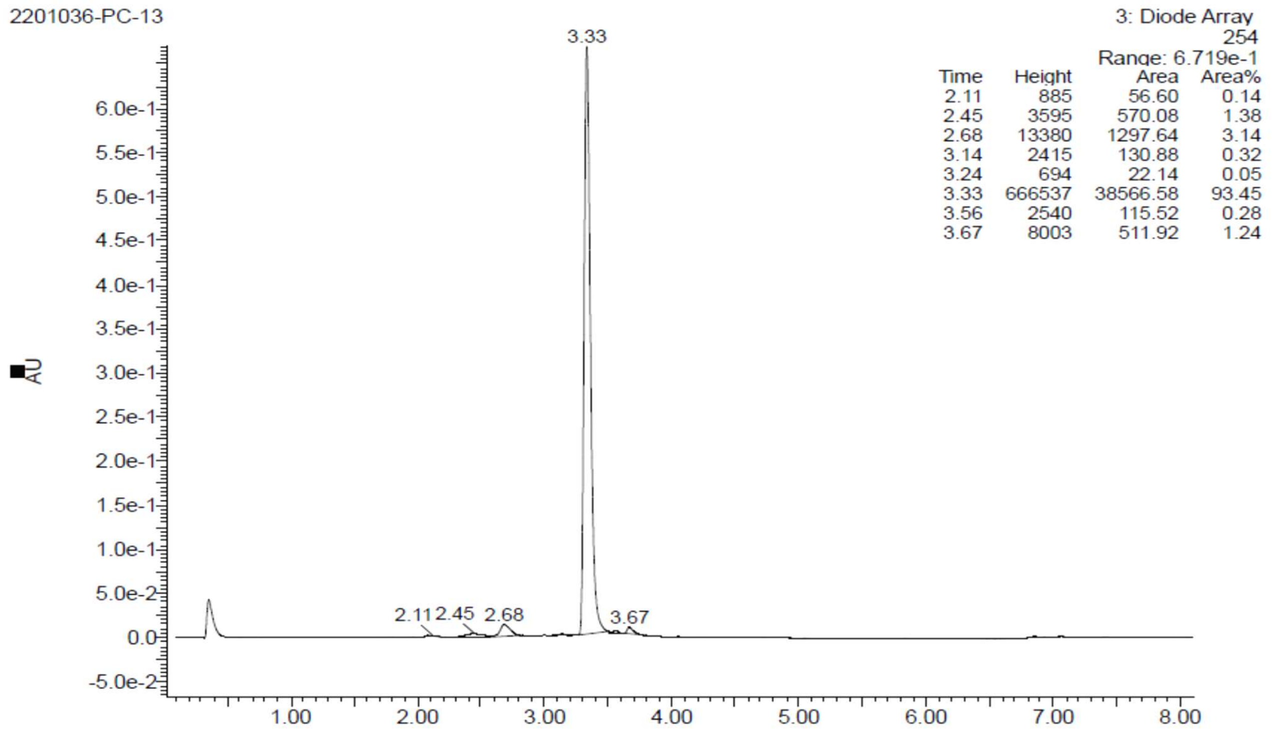

LC of PC-13 (5e)

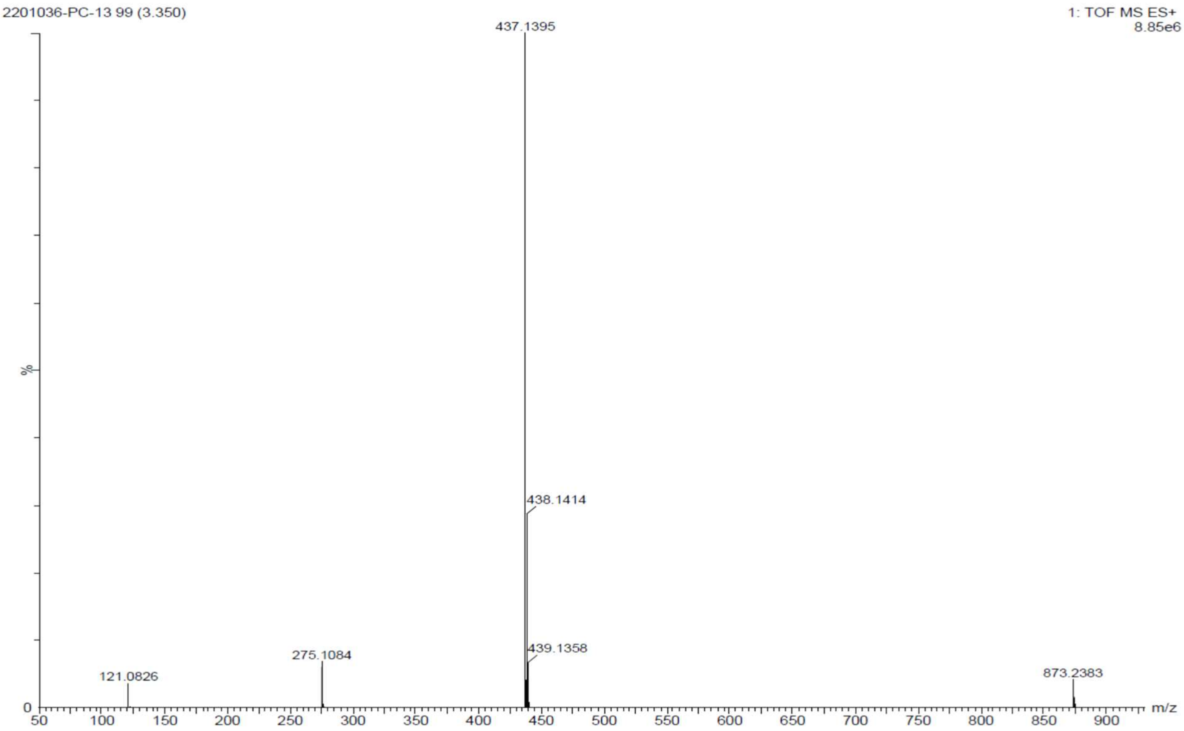

Mass spectrum of PC-13 (5e)

- **Cell line: MCF7 (2000 cells/per well[96])**
- **Treated time: 72hrs**
- **Assay: alamarBlue (4hrs incubated)**
- **Data: PC-13**

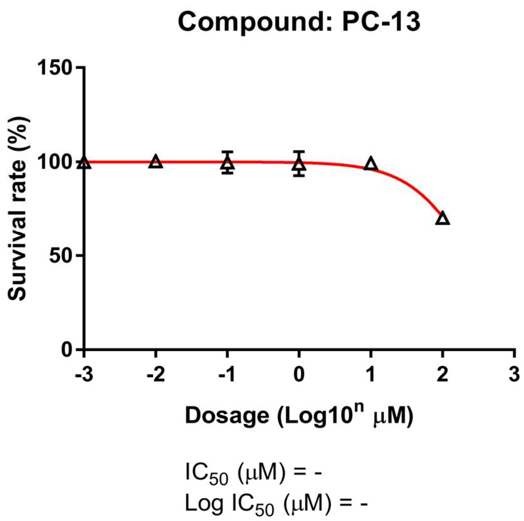

Conc. (μM)	Viability	
	AVE.	± SD.
**0**	100.00	2.10
**0.01**	100.36	0.48
**0.1**	99.71	5.65
**1**	99.05	6.44
**10**	99.35	2.87
**100**	70.32	3.03

Log curve of PC-13 (5e)

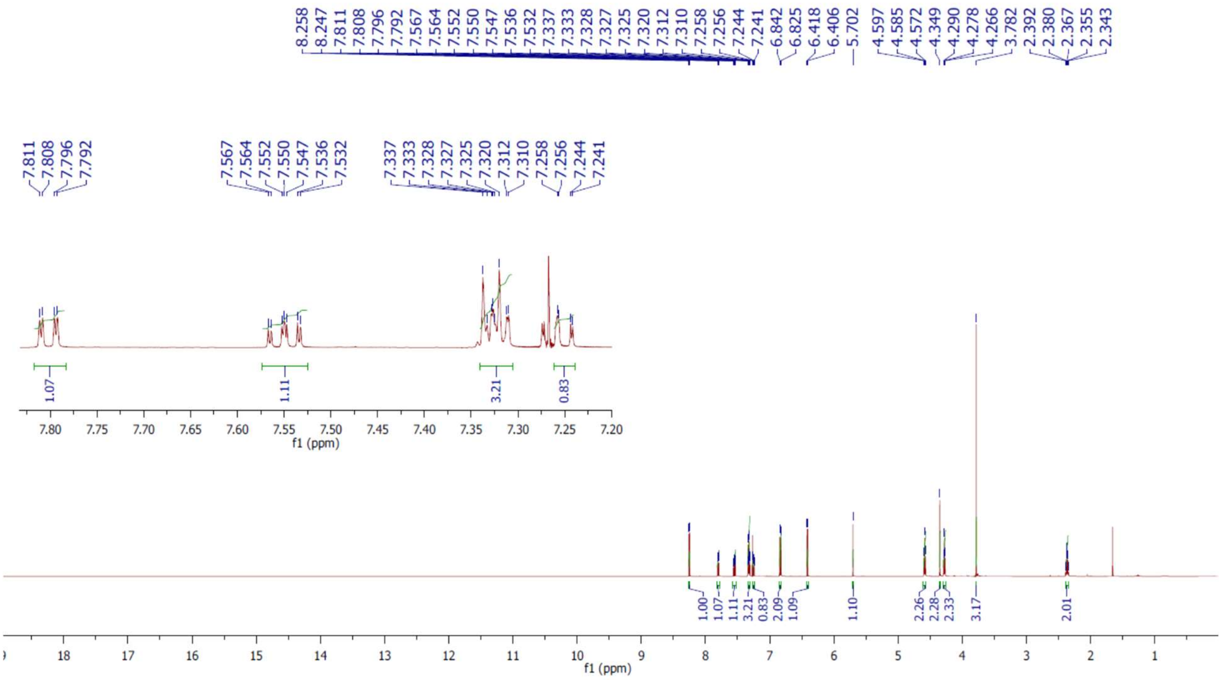

¹H NMR spectrum of PC-14 (5f)

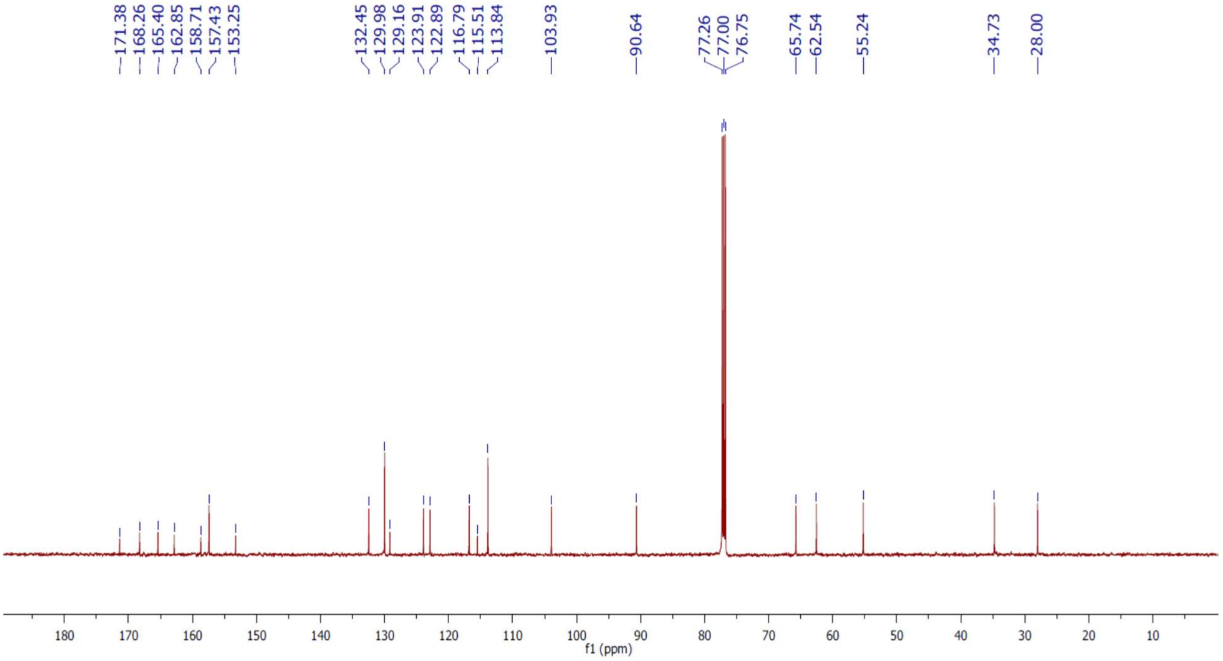

¹³C NMR spectrum of PC-14 (5f)

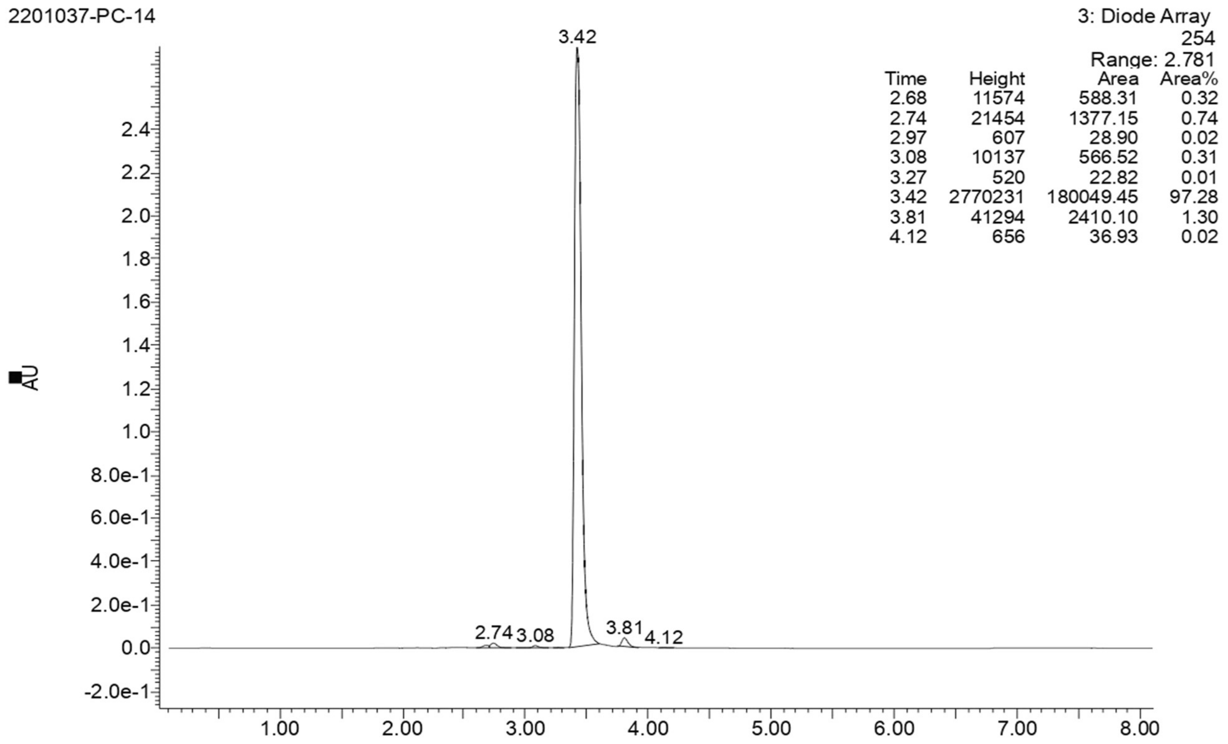

LC of PC-14 (5f)

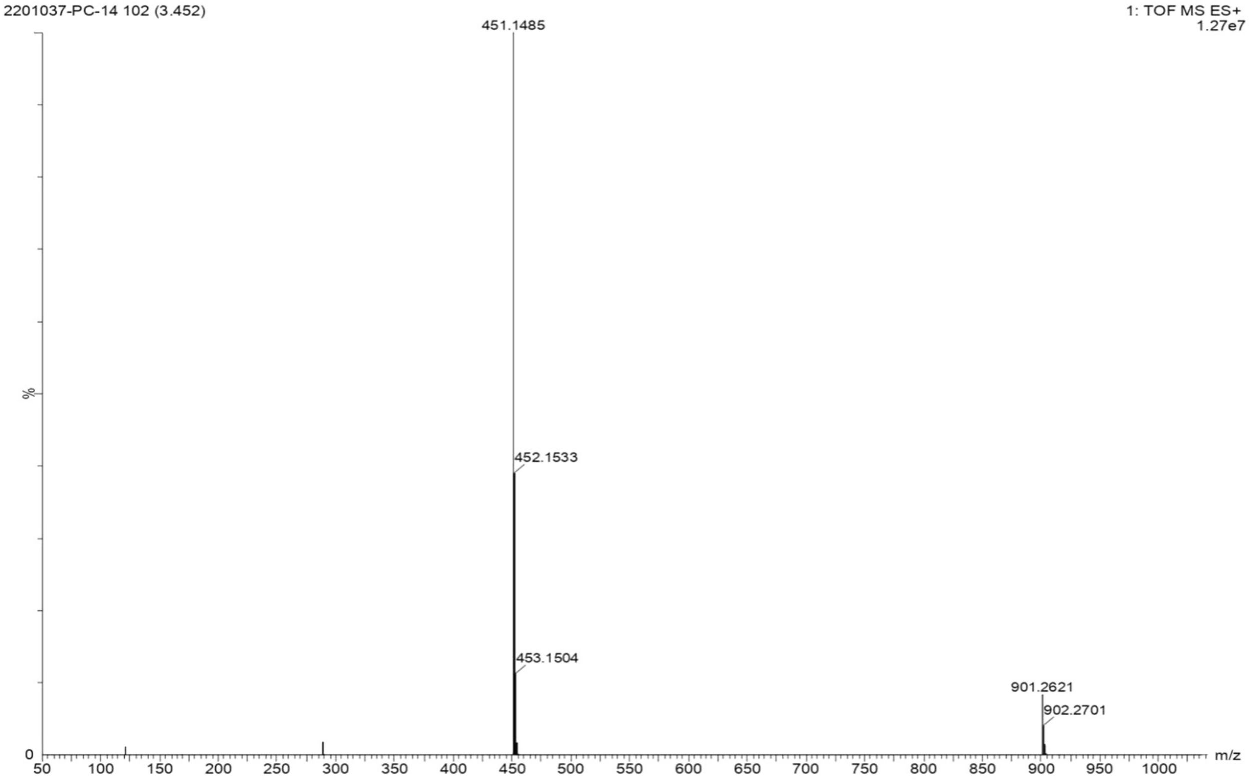

Mass spectrum of PC-14 (5f)

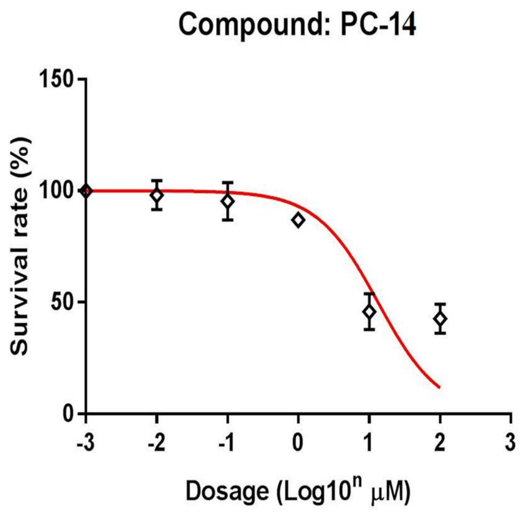

IC_{50} (μM) = 13.33
Log IC_{50} (μM) = 1.13

- Cell line: MCF7 (2000 cells/per well[96])
- Treated time: 72hrs
- Assay: alamarBlue (4hrs incubated)
- Data: PC-14

Conc. (μM)	Viability	
	AVE.	± SD.
**0**	100.00	2.10
**0.01**	98.02	6.55
**0.1**	95.27	8.47
**1**	86.95	2.84
**10**	45.76	8.04
**100**	42.70	6.55

Log curve of PC-14 (5f)

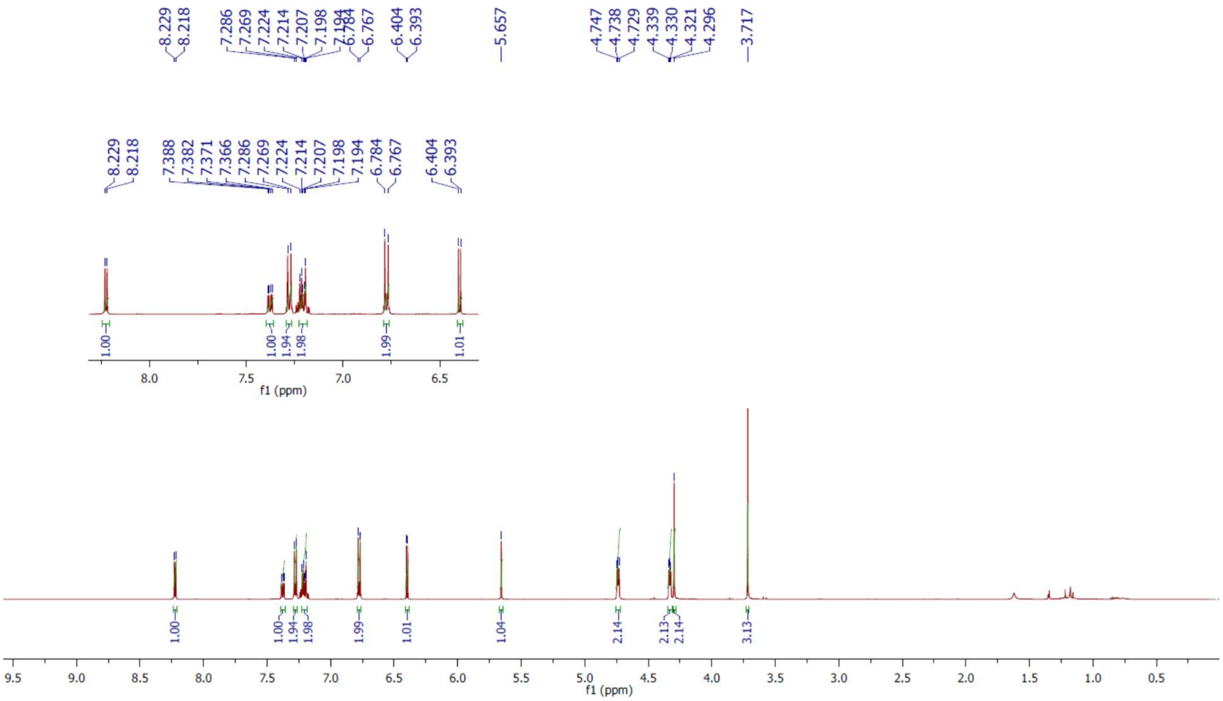

^{1}H NMR spectrum of PC-15 (5g)

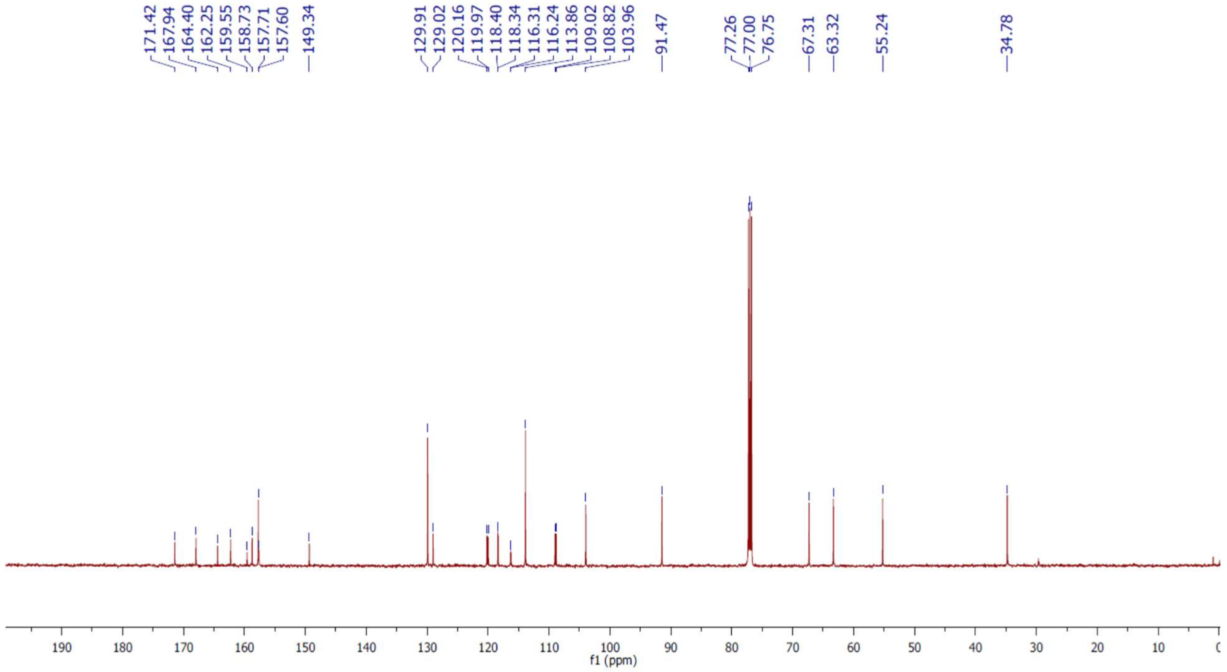

13C NMR spectrum of PC-15 (5g)

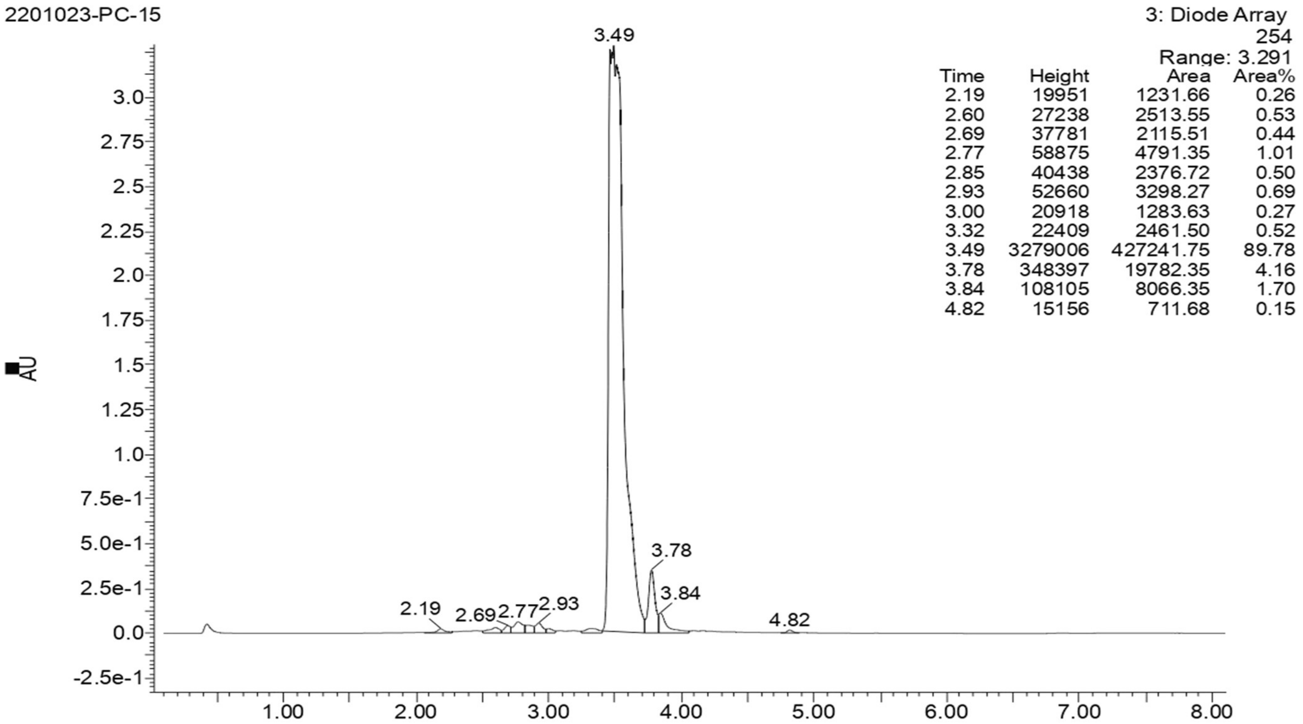

LC of PC-15 (5g)

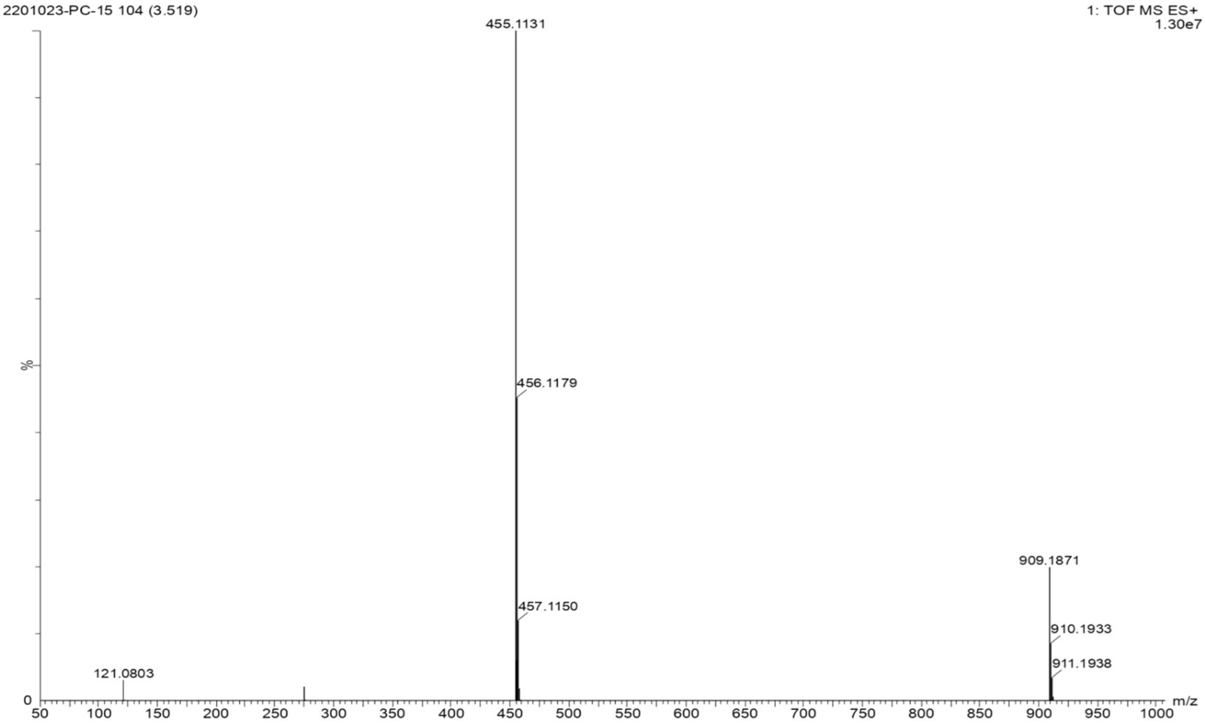

Mass spectrum of PC-15 (5g)

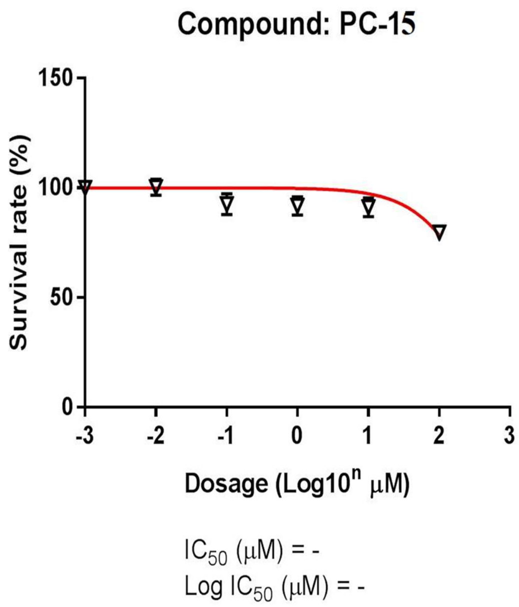

- Cell line: MCF7 (2000 cells/per well[96])
- Treated time: 72hrs
- Assay: alamarBlue (4hrs incubated)
- Data: PC-15

Conc. (μM)	Viability	
	AVE.	± SD.
0	100.00	2.10
0.01	100.32	3.63
0.1	92.57	4.74
1	91.82	4.19
10	91.19	4.32
100	79.66	2.96

Log curve of PC-15 (5g)

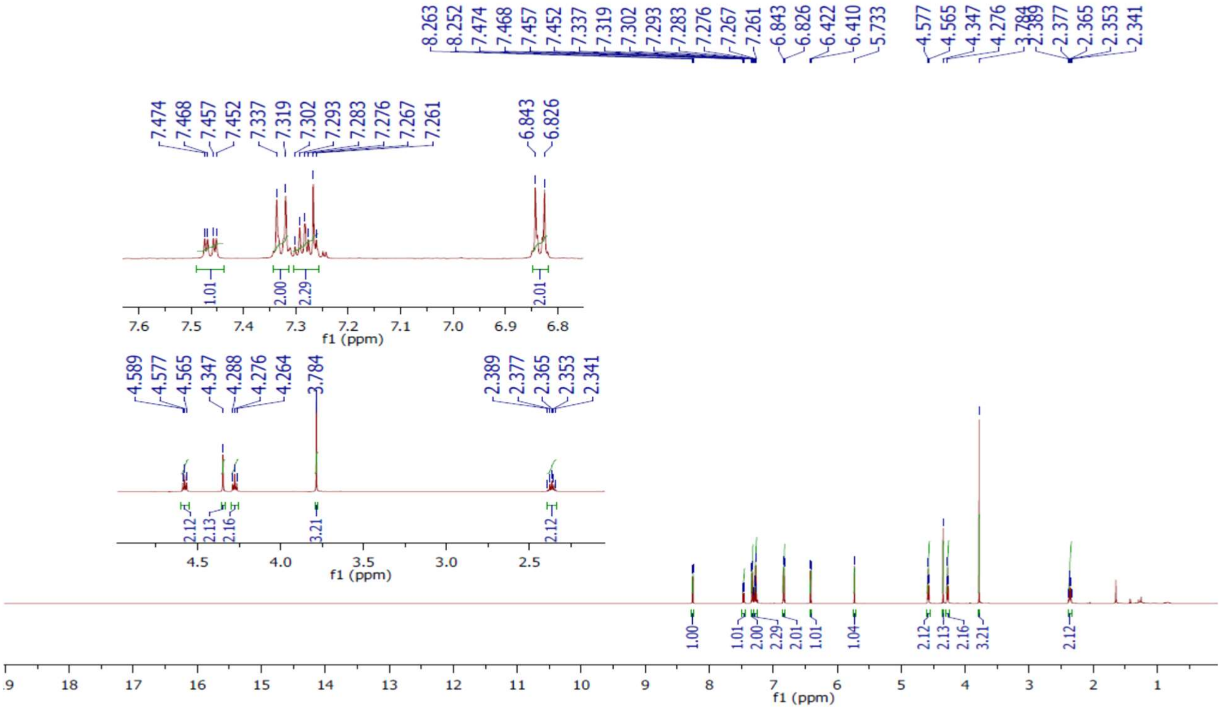

^{1}H NMR spectrum of PC-16 (5h)

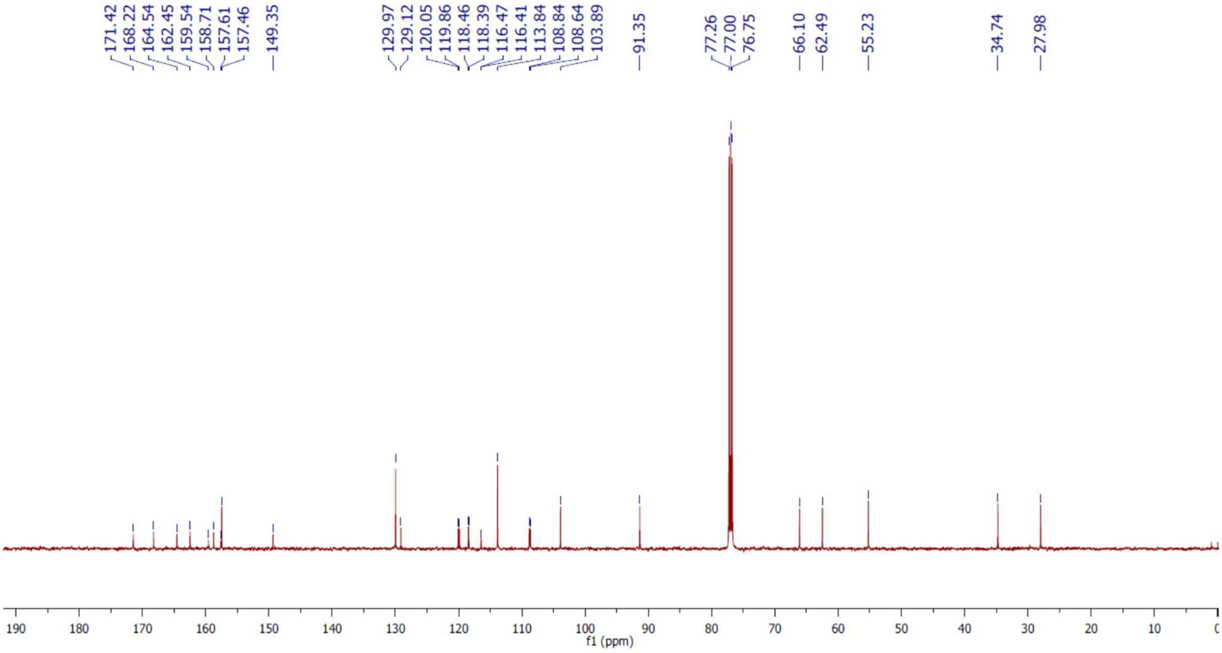

^{13}C NMR spectrum of PC-16 (5h)

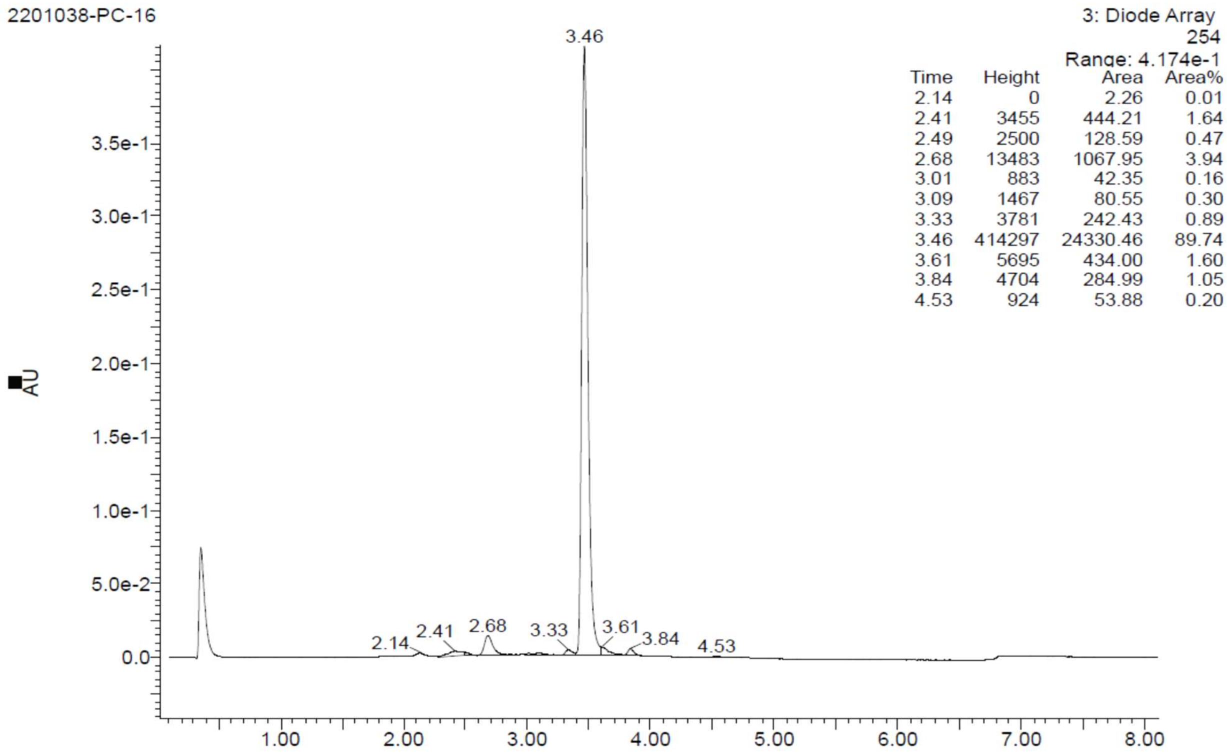

LC of PC-16 (5h)

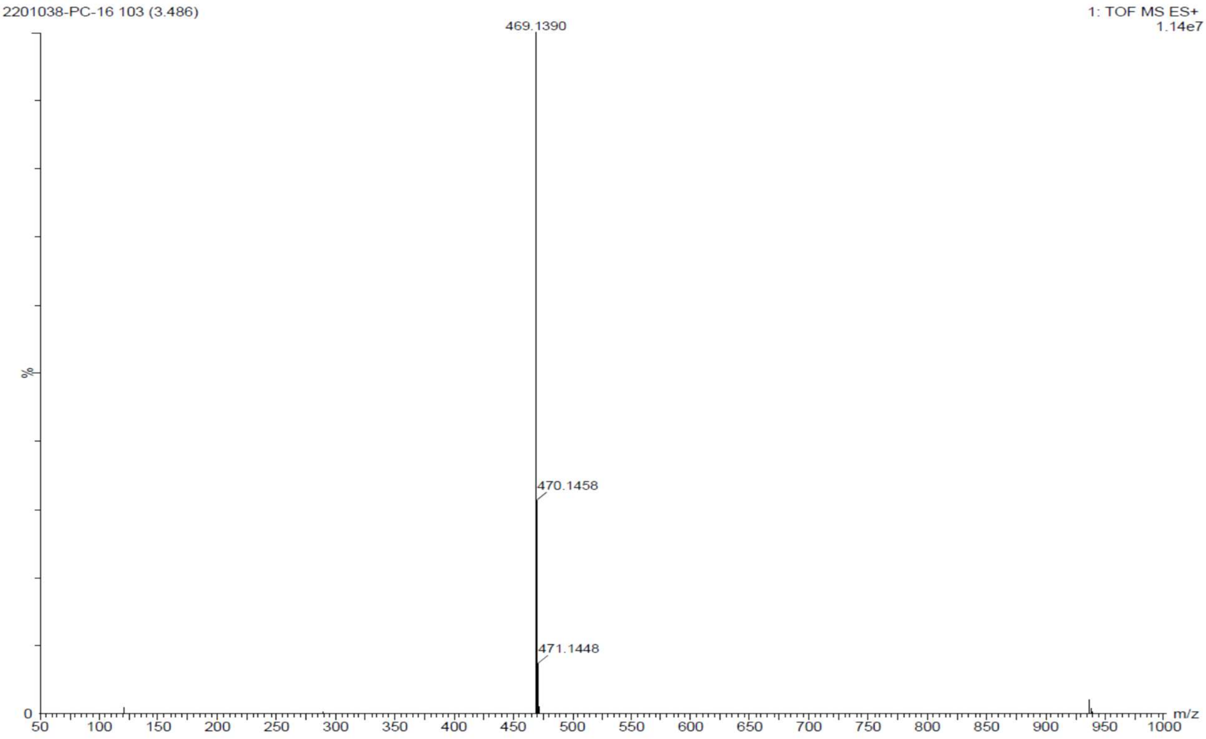

Mass spectrum of PC-16 (5h)

- Cell line: MCF7 (2000 cells/per well[96])
- Treated time: 72hrs
- Assay: alamarBlue (4hrs incubated)
- Data: PC-16

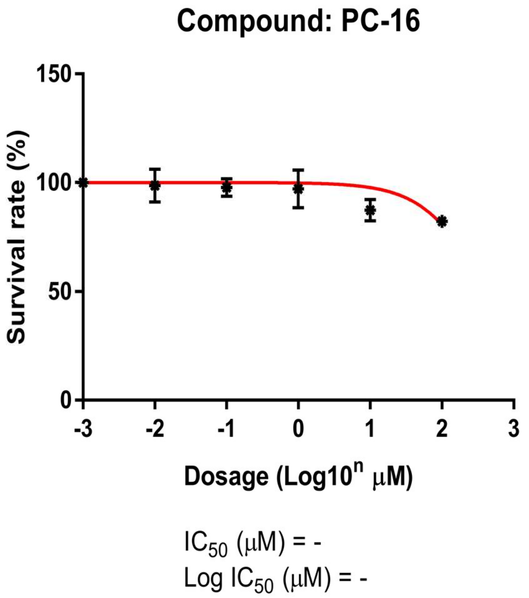

Conc. (μM)	Viability	
	AVE.	± SD.
0	100.00	2.10
0.01	98.59	7.47
0.1	97.69	3.99
1	97.01	8.67
10	87.30	4.93
100	82.21	0.81

Log curve of PC-16 (5h)
